# Extravesicular chloride ion regulates the uptake of ATP, sulfate, and inorganic phosphate by membrane vesicles from bovine adrenal chromaffin granules

**DOI:** 10.1016/j.jbc.2025.110892

**Published:** 2025-11-04

**Authors:** Yoshinori Moriyama, Seiji Nomura, Sawako Moriyama, Nao Hasuzawa, Masatoshi Nomura

**Affiliations:** 1 Division of Endocrinology and Metabolism, Department of Internal Medicine, Kurume University School of Medicine, Fukuoka, Japan; 2 Department of Medical Biochemistry, Kurume University School of Medicine, Fukuoka, Japan

**Keywords:** chloride ion, chromaffin granule, inorganic phosphate, phosphoenolpyruvate, SLC17A9, sulfate, vacuolar H^+^-ATPase, VNUT

## Abstract

ATP stored in secretory vesicles is released into the extracellular space through exocytosis, acting as an intercellular messenger in purinergic signaling. The vesicular nucleotide transporter (VNUT) is crucial for filling the vesicles with ATP. However, the mechanisms that regulate this transport are not yet fully understood. This study explores how anions influence ATP uptake in membrane vesicles from the bovine adrenal chromaffin granules. Our findings indicate that ATP uptake is driven by the membrane potential established by the vacuolar H^+^-ATPase and is sensitive to diisothiocyanostilbene disulfonic acid, phosphoenolpyruvate, atractyloside, and 2′(or-3′)-*O*-(*N*-Methylanthraniloyl) adenosine 5′-triphosphate (MANT-ATP). Notably, extravesicular Cl^-^ significantly influences the ATP uptake: facilitation of ATP uptake occurs at millimolar Cl^-^ concentrations, peaking between 5 to 20 mM, although it declines sharply at higher Cl^-^ levels. A considerable amount of ATP is taken up by nigericin plus K^+^ without Cl^-^. The membrane vesicles took up radiolabeled Cl^-^ in an ATP- and membrane potential-dependent fashion, which is partially sensitive to 5-nitro-2-(3-phenylpropylamino)benzoic acid and MANT-ATP but not to AMP. Sulfate and inorganic phosphate (Pi) at mM levels inhibit ATP uptake and are taken up in an ATP-dependent manner, exhibiting similar energetics, Cl^-^ dependency, and pharmacological profile to ATP uptake. In the mouse adrenal gland membranes, the uptake of sulfate and Pi depended on ATP, while this is not the case for those from VNUT knockout mice. These results suggest that VNUT transports not only nucleotides but also inorganic oxyanions, utilizing Cl^-^ as a regulatory factor rather than an essential component of its activity.

ATP, the energy currency in cellular metabolism and motility processes, also acts as an intercellular messenger, at least in the animal kingdom: ATP is stored in secretory organelles and released by exocytosis upon stimulation. Then, ATP and its degradation products, ADP and adenosine, bind to purinoceptors, triggering post-cellular responses in various pathophysiological processes, such as pain transmission and the development of inflammation. Thus, vesicular ATP filling is an event that indicates when, where, and how purinergic chemical transmission is initiated and is crucial for understanding the pathophysiological processes involved (1, 2, 3, 4, 5, 6, 7, 8).

Vesicular ATP filling occurs through the active transport of ATP by the VNUT into ATP-filled granules (8, 9). VNUT, which is the ninth member of the SLC17 organic anion transporter family (SLC17A9), is located in ATP-filled granules such as adrenal chromaffin granules, insulin granules in islet β cells, and brain synaptic vesicles (8, 9, 10, 11, 12, 13). When VNUT expression is suppressed, the concentration of vesicular ATP decreases, leading to a reduction or complete loss of ATP secretion from purinergic cells, depending on the level of suppression (9, 14, 15). Mice with a knockout of the *SLC17A9* gene exhibit normal growth compared to wild-type mice. However, these knockout mice consistently show impaired purinergic chemical transmission, which reduces the secretion of inflammatory cytokines such as IL-6, improves insulin sensitivity, and diminishes pain sensation (16, 17). Additionally, *SLC17A9* gene knockout mice demonstrate significant improvements in inflammatory cell infiltration and fibrosis in a model of high-fat diet-induced non-alcoholic steatohepatitis (NASH) (13, 18). In contrast, overexpression of VNUT in neurons leads to increased ATP secretion, resulting in hyperalgesia and chronic inflammation (19). There is also a reported positive correlation between *SLC17A9* gene expression levels and the malignancy of various cancers, including liver and lung cancers (20, 21, 22, 23). The administration of VNUT inhibitors, such as clodronate and Evans blue, to mice reduces ATP secretion by decreasing vesicular ATP filling. This alleviates chronic neuropathic pain, as well as pain induced by complete Freund’s adjuvant, carrageenan, and β-glucan (12, 13, 17, 18, 24, 25, 26). Moreover, clodronate significantly improved activity scores in NASH, along with reducing inflammatory cell infiltration and liver fibrosis in a methionine choline-deficient diet-induced NASH model (27). These findings indicate that VNUT is an important target for developing treatments for intractable and metabolic disorders and understanding its transport mechanisms and regulation is a significant medical issue, although the details remain poorly understood.

One fascinating aspect of VNUT research is its interaction with anions. The SLC17 family of organic anion transporters is active at low concentrations of Cl^-^ and Br^-^, specifically in millimolar levels (28, 29, 30, 31). Initial biochemical studies with isolated synaptic vesicles and synaptic-like microvesicles demonstrated that adding glutamate and ATP to a buffer containing 4 mM Cl^-^ resulted in the uptake of H^+^ into the vesicles (32, 33, 34, 35). These findings suggested that Cl^-^ was transported simultaneously with glutamate, providing indirect evidence of Cl^-^ conductance. Further observations showed that when NPT1 (SLC17A1 protein) was expressed in *Xenopus* oocytes, Cl^-^ conductance was detected (36). This characteristic has been extensively studied at the molecular level, particularly for vesicular glutamate transporters (SLC17A6-8, VGLUTs) (28, 29, 30, 31, 37, 38, 39, 40). Consequently, it became clear that while Cl^-^ is not essential for glutamate transport, it plays a crucial regulatory role in modulating glutamate transport activity in conjunction with the synaptic vesicle cycle in nerve terminals (28, 29, 30, 31). Meanwhile, using purified VNUT-containing proteoliposomes, we found that VNUT exhibits a Cl^-^ activation effect similar to that of VGLUT (9). However, the activation patterns differ significantly from ATP uptake in the membrane vesicles of adrenal chromaffin granules (CHRMVs) (41). For instance, the optimal Cl^-^ concentration for activation in VNUT-containing proteoliposomes was approximately 5 mM, whereas for CHRMVs, it was around 50 mM, reflecting a tenfold difference. The cause of this discrepancy and whether it reflects VNUT's function in ATP-filled granule remains unclear, especially since research on ATP uptake in ATP-filled granules has stagnated for the past 30 years.

In this study, we aimed to address the issues by analyzing the effect of anions on ATP uptake using CHRMVs. Our findings suggest that VNUT can transport sulfate and Pi alongside nucleotides and that Cl^-^ is not essential but acts as a regulator of these transport cycles.

## Results

### ATP-dependent ATP uptake is energetically coupled with V-ATPase

ATP hydrolysis and subsequent formation of a proton motive force by V-ATPase are required to initiate ATP transport into vesicles. Following a report by Bankston and Guidotti (41), an ATP-regenerating system consisting of creatine phosphate and creatine kinase was added to the assay mixture. This addition helps maintain the concentration of the transport substrate and minimizes the depletion of ATP by V-ATPase and other ATP-consuming proteins, ensuring accurate measurement of the transport process.

**Figure 1:**
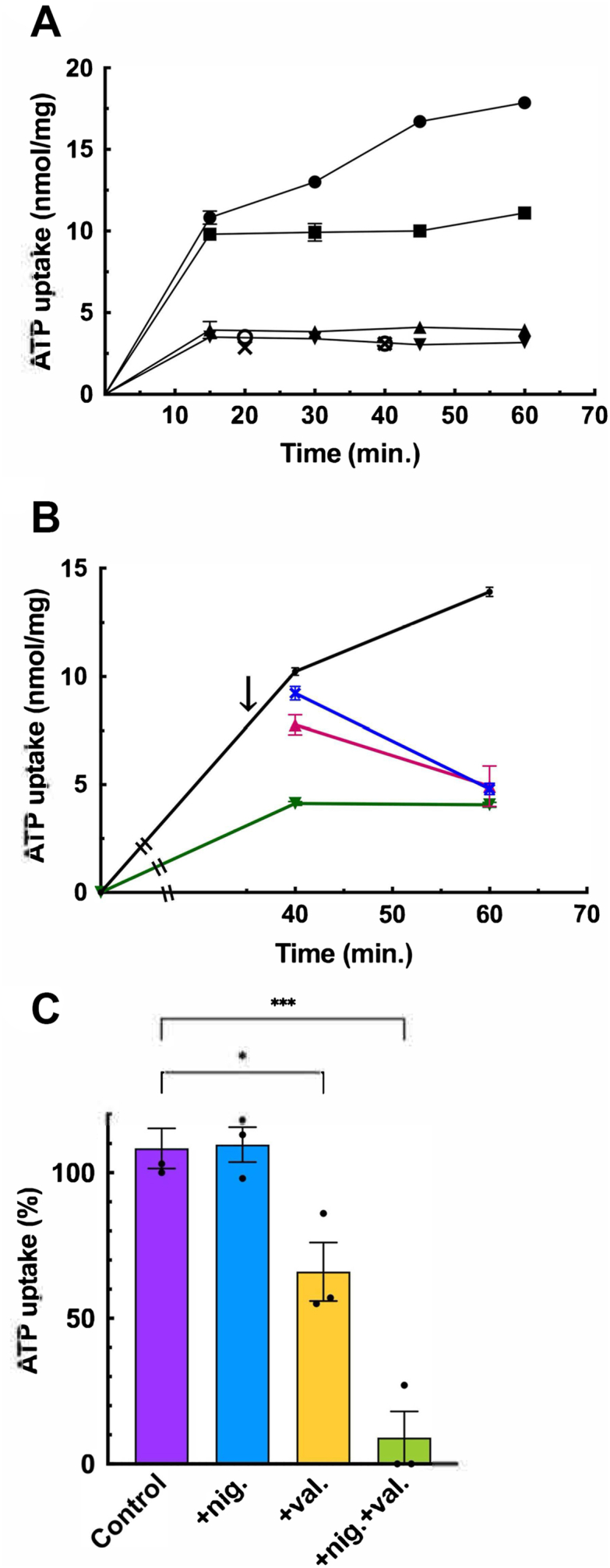
**ATP uptake by CHRMVs**. *A*, time course of ATP uptake in the presence (● ▲ ▼ X) and absence (■) of the ATP-regenerating system. Additions; ConB (▼) CCCP (▲), ConB + CCCP (X), Bafilomycin (Baf) (○). n = 3. Data represent means ± SEM. Statistical analysis was done using Student’s *t* test. *B*, release of ATP from CHRMVs. As indicated by an arrow, the following compounds were added: con B (▲) and CCCP (▼). n = 3. *C*, energetics. ATP uptake by K-acetate-trapped CHRMVs was assayed in the buffer containing 20 mM MOPS-tris, pH 7.0, 0.1 M K-acetate, 5 mM KCl, 0.2 M sucrose, 5 mM Mg acetate, 1 mM [^3^H] ATP, and ATP-regenerating systems in the absence (Control) or presence of valinomycin (val) at 0.1 μg or nigericin (nig) at 0.1 μg or both at 0.1 μg each for 30 min. Total (100%) activity corresponded to 12.8 nmol/mg protein. n = 3. Data represent means ± SEM. ∗*p* = 0.220, ∗∗∗*p* < 0.0001 Statistical analysis was done using Student’s *t* test.

As shown in Figure 1*A* and Figure S1, CHRMVs took up radioactive ATP in a time-dependent fashion. Without an ATP regenerating system, the uptake rate was little increased. Adding bafilomycin A1, a V-ATPase inhibitor (42, 43), inhibited ATP uptake by approximately 60%. Another V-ATPase inhibitor, concanamycin B (con B) (44), and uncoupler CCCP gave similar results. The effects of con B and CCCP were not additive. Furthermore, either con B or CCCP after 35 min of incubation with ATP released ATP taken up by the membrane vesicles (Fig. 1*B* and S2). The results indicate that the proton motive force generated by V-ATPase is essential for both bafilomycin A1-and con B-sensitive ATP uptake and its maintenance within the vesicles. Previous studies have shown that the addition of valinomycin, an electrogenic K^+^ ionophore, dissipates the membrane potential (Δψ, inside positive) and increases the pH gradient (ΔpH, inside acidic) by increasing the membrane permeability to K^+^ in the chromaffin granule membrane. In contrast, nigericin, an electroneutral H^+^/K^+^ antiport ionophore, dissipates the ΔpH (inside acidic) without significantly affecting or increasing Δψ (inside positive) (45, 46, 47, 48). As shown in Figure 1*C*, the addition of valinomycin partially inhibited ATP uptake, while nigericin had no significant impact on ATP uptake. Additionally, when both valinomycin and nigericin are added, they dissipate both ΔpH and Δψ (45, 46, 47, 48), almost completely inhibiting ATP uptake, similar to the effects observed with either CCCP or con B (Fig. 1*C*). These results indicate that Δψ established by V-ATPase is a dominant driving force of bafilomycin A1-or con B-sensitive ATP uptake. From now on, con B-sensitive ATP-dependent ATP uptake is defined as ATP uptake.

### Extravesicular Cl^-^ regulates ATP uptake

**Figure 2:**
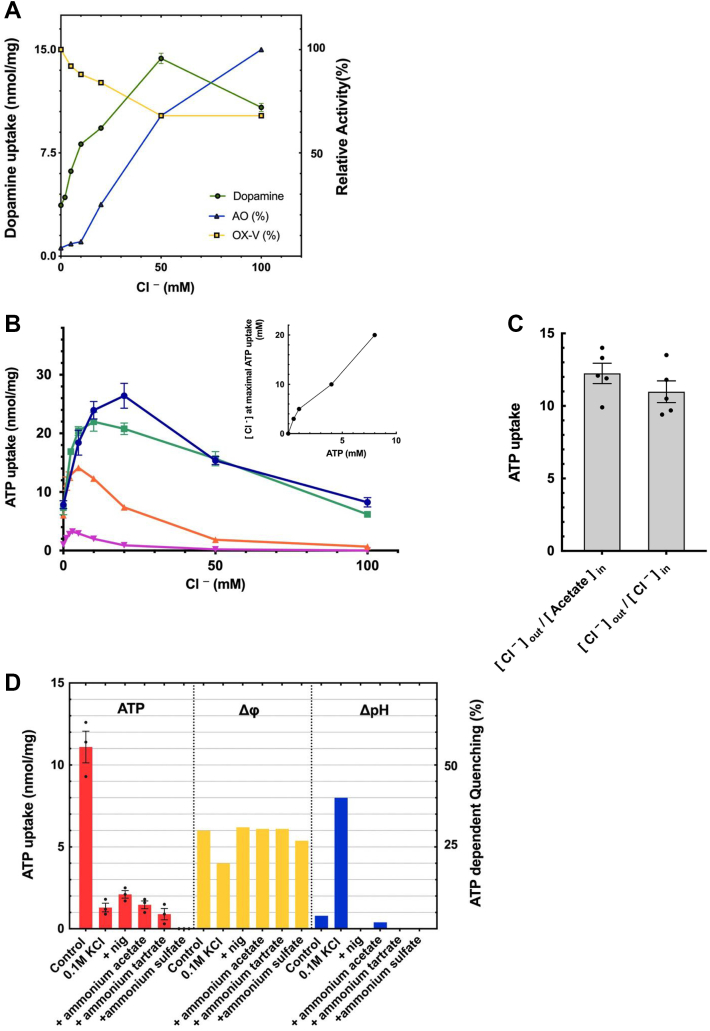
**Effect of Cl^-^ on the ATP uptake**. *A*, KCl dose dependence on ATP-dependent dopamine uptake, formation of Δψ, and ΔpH. ATP-dependent uptake of [^3^H]dopamine (●) and formation of ΔpH (▲), Δψ (□) by CHRMVs were measured in the assay mixture containing various KCl concentrations. Total (100%) control corresponded to 30 and 45% of acridine orange and oxonol-V quenchings, respectively. Dopamine uptake in the absence of ATP corresponds to 0.3 nmol/mg n = 3. Data represent means ± SEM. Statistical analysis was done using Student’s *t* test. The magnitude of quenching indicates the relative increase in ΔpH and Δψ associated with H^+^ - transport. Formation of both ΔpH and Δψ in the absence of ATP was negligible (n = 1). *B*, KCl dose dependence on ATP uptake by CHRMVs was carried out under the standard assay condition, except that the concentrations of KCl and [^3^H] ATP in the assay mixture were as indicated. The ATP concentration 0.5 mM (▼, *magenta*); 1 mM (▲, *orange*); 4 mM (■, *green*); 8 mM (●, *purple*). (*Inset*) The Cl^-^ concentration giving the maximum ATP uptake activity at each substrate concentration was obtained from Figure 2 B and plotted. n = 3, Data represent means ± SEM. Statistical analysis was done using Student’s *t* test. *C*, internal Cl^-^ does not affect ATP uptake. CHRMVs trapped with 10 mM KCl or 10 mM K-acetate were prepared according to the *EXPERIMENTAL PROCEDURES*, and their ATP-dependent ATP uptake was measured as described in the legend of Figure 1 in the buffer containing 5 mM KCl. n = 5, Data represent means ± SEM. Statistical analysis was done using Student’s *t* test. *D*, ATP uptake and ATP-dependent formation of Δψ and ΔpH were measured in the assay mixture containing either 5 mM KCl (*control*) or 0.1 M KCl as indicated. Assays in the presence of KCl at 0.1 M and ammonium salts at 10 mM, or nigericin (nig) at 0.1 μg, as indicated, were also performed. ATP uptake, n = 3. Data represent mean ± SEM. Statistical analysis was performed using Student’s *t* test. Measurements of Δψ and ΔpH, n = 1.

Then, we investigated the effect of Cl^-^ in ATP uptake. It is widely accepted that the transport of H^+^ by V-ATPase in chromatin granules is dependent on Cl^-^, and the ΔpH generated increases with higher Cl^-^ concentrations, while the Δψ produced by V-ATPase peaks in the absence of Cl^-^ and decreases as Cl^-^ concentrations increase (28, 45). As a control experiment, we first measured the degree of ΔpH and Δψ formation, and the magnitude of dopamine transport when the V-ATPase was activated in the presence or absence of varying Cl^-^ concentrations. As shown in Figure 2*A* and Figure S3, upon adding ATP, V-ATPase-driven electrogenic H^+^ transport formed an Δψ (inside positive) and ΔpH (inside acidic) across the membrane, as revealed with fluorescence quenching of oxonol-V and acridine orange (45, 46, 49, 50), respectively. ATP-dependent formation of Δψ decreased with increasing Cl^-^ concentration, but approximately 70% remained even at 0.1 M Cl^-^. In contrast, ΔpH was only slightly detectable in the absence of Cl^-^ but increased rapidly from 5 mM and reached a maximum at 0.1 M Cl^-^, consistent with the previous observations (45, 46, 49, 50). Furthermore, because ATP-dependent monoamine transport is a VMAT-mediated exchange with 2H^+^ and monoamine (51, 52), ATP-dependent dopamine uptake was approximately 15% of the maximum activity in the absence of Cl^-^, increased with increasing Cl^-^ concentration (up to 50 mM), and decreased slightly at 0.1 M, showing ΔpH is far more dominant than Δψ as the driving force for dopamine transport (Fig. 2*A*).

Subsequently, we measured the ATP uptake in the presence or absence of varying Cl^-^ concentrations. As shown in Figure 2*B* and Figure S4, the ATP uptake was strongly enhanced by Cl^-^ at the mM level. Under standard assay conditions (1 mM ATP, 5 mM Mg^2+^), the Cl^-^ concentration showing maximal uptake was approximately 5 mM. The concentration of Cl^-^ required for maximum activation of ATP uptake depended on the ATP concentration in the assay system and increased almost linearly up to 8 mM (Fig. 2*B inset*). Activation required extravesicular Cl^-^, which was independent of intravesicular Cl^-^ (Fig. 2*C*). The pattern of increased activity due to Cl^-^ closely mirrored that of VNUT-containing proteoliposomes (9).

At higher concentrations of Cl^-^, the ATP uptake decreased and became almost zero at concentrations of over 50 mM (Fig. 2*B*). The reduction in activity at high Cl^-^ concentrations resembles what is observed in ATP-dependent glutamate transport within synaptic vesicles (53), synaptic-like microvesicles of pineal glands (34), and in proteoliposomes reconstituted with purified VGLUT2 and F-ATPase (54). It is unlikely that the rapid reduction in ATP uptake observed at high Cl^-^ concentrations is due to a decrease in Δψ, as the change in Δψ was insignificant when compared to the degree of reduction in ATP uptake (Fig. 2*D*). Furthermore, once ATP uptake was reduced, it did not recover even after adding ammonium acetate, ammonium tartrate, or nigericin to convert the resulting H^+^ concentration to Δψ (Fig. 2*D*). Together, these findings suggest that extravesicular Cl^-^ regulates ATP uptake, while neither an internal acidic pH nor Δψ directly influences the Cl^-^ effect at high concentrations on the reduced ATP uptake. The possible mechanism of the reduction will be discussed later.

### Cl^-^ is not essential for ATP uptake

We noticed that CHRMVs accumulate appreciable amounts of ATP even in the absence of Cl^-^ (Fig. 2*A*). We investigated the properties of this ATP uptake, namely Cl^-^-independent ATP uptake.

**Figure 3:**
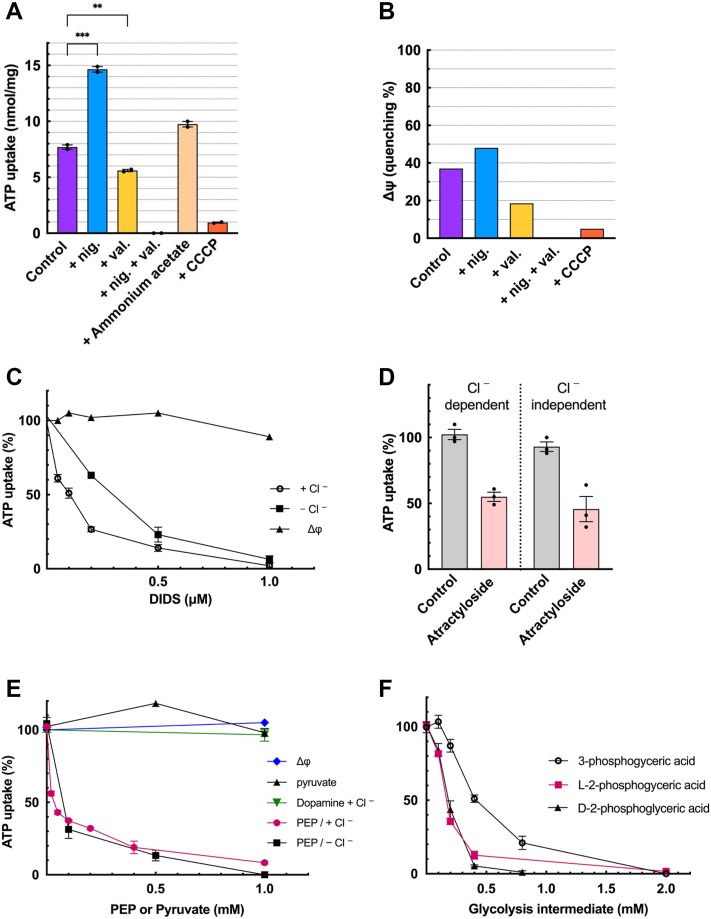
**Characterization of Cl^-^-independent ATP uptake**. *A*, ATP uptake by K-acetate-loaded CHRMVs in the absence of Cl^-^. Uptake of [^3^H]ATP by K-acetate trapped CHRMVs was carried out according to the *EXPERIMENTAL PROCEDURES* in the assay mixture consisting of 20 mM MOPS-tris, pH 7.0, 0.1 M K-acetate, 0.1 M sucrose, 5 mM Mg-acetate, 1 mM [^3^H]ATP and ATP regenerating systems. ATP uptake at 40 min in the presence of con *B* was 1 μM was subtracted. Additions: none, nigericin, valinomycin, CCCP at 0.5 μM each; ammonium acetate 5 mM. n = 3. Data represent means ± SEM. Statistical analysis was performed using Student’s *t* test. *B*, ATP-dependent formation of Δψ in K-acetate trapped CHRMVs as revealed with oxonol-V fluorescence quenching. The assay medium contained 20 mM MOPS-tris, pH 7.0, 0.1 M K-acetate, 0.1 M sucrose, 5 mM Mg-acetate, 5 μM oxonol-V, and 10 to 15 μg membrane vesicles in the absence (control) or presence of nigericin (nig) at 0.1 μg, valinomycin (val) at 1 μg, or con *B* at 1 μM. After incubation at room temperature for approximately 5 min, ATP (1 mM) was added and the maximum quenching was plotted. n = 1. *C*, The effect of DIDS on Cl^-^ -dependent(○) and independent(■)ATP uptake was measured according to the *EXPERIMENTAL PROCEDURES* at the listed DIDS concentrations. Total activities (100%) of Cl^-^-dependent and independent ATP uptake correspond to 12.5 and 7.8 nmol/mg protein at 40 min, respectively. n = 3. Data represent mean ± SEM. Statistical analysis was performed using Student’s *t* test. ATP-dependent formation of Δψ at the indicated DIDS concentrations is also shown. 100% corresponded to 19% of quenching. n = 1. *D*, The effect of atractyloside on Cl^-^-dependent and -independent ATP uptake was measured as described above in the absence or presence of atractyloside 50 μM. Full activity (100%) corresponded to 10 nmol ATP/mg protein at 30 min n = 3. Data represent means ± SEM. Statistical analysis was performed using Student’s *t* test. *E*, The effect of PEP on Cl^-^-dependent and -independent ATP uptake at 30 min was measured as described above in the absence (100%) or presence of indicated PEP concentrations. ATP-dependent dopamine uptake at 15 min and formation of Δψ was also shown. Full activity (100%) of Cl^-^-dependent and -independent ATP uptake and dopamine uptake corresponded to 10 and 6.9 nmol ATP/mg protein, 13 nmol dopamine/mg protein. n = 3. Data represent means ± SEM. Statistical analysis was performed using Student’s *t* test. 100% Δψ formation corresponded to 25% quenching. n = 1. *F*, The effect of some glycolysis intermediates on Cl^-^-independent ATP uptake. The assay was performed as described above in the absence or presence of D(−)-3-phosphoglyceric acid (○), D(+)2-phosphoglyceric acid (▲) or L-2-phosphoglyceric acid (■) at the indicated concentrations. Full activity (100%) corresponded to 14.5 nmol ATP/mg protein at 40 min n = 3. Data represent means ± SEM. Statistical analysis was performed using Student’s *t* test.

As shown in Figure 3*A*, the ATP uptake by K-acetate-trapped CHRMVs was measured in a buffer containing 0.1 M K-acetate. Adding nigericin enhanced ATP uptake. This value was comparable to, but not as high as, the maximum ATP uptake in the presence of Cl^-^ (see Fig. 2*B*). Cl^- _^independent ATP uptake was reduced by valinomycin and almost completely abolished by adding nigericin plus valinomycin or CCCP. Adding ammonium acetate to convert ΔpH to Δψ did not recover ATP uptake. Under this condition, Δψ produced by V-ATPase behaved similar to ATP uptake, suggesting that Cl^-^-independent ATP uptake is also driven by Δψ (Fig. 3*B*). Pharmacological analysis was performed to elucidate the differences in the properties of Cl^-^-independent and -dependent ATP uptake. DIDS irreversibly inhibits VNUT at sub-μM levels (9). Both Cl^-^-independent and -dependent ATP uptakes were strongly inhibited by DIDS (Figs. 3*C* and S5). In this concentration range, DIDS was ineffective against Δψ formation by V-ATPase and ATP-dependent dopamine uptake by VMAT. Next, we investigated the effects of the ATP/ADP exchanger inhibitor atractyloside and the glycolytic intermediate PEP. The former has been reported to inhibit VNUT (9), and the latter to inhibit ATP uptake in chromaffin granules (55). Both, like DIDS, inhibited ATP uptake regardless of the presence or absence of Cl^-^ (Fig. 3, *D* and *E* and Fig. S6). In particular, the ID50 of PEP was approximately 20 μM, regardless of the presence or absence of Cl^-^. On the other hand, PEP at 1 mM was ineffective against both ATP-dependent dopamine transport and V-ATPase-evoked Δψ formation (Fig. 3*E*). The structural analog pyruvate had no effect even at 1 mM. These results indicated that Cl^-^-independent ATP uptake exhibits energetics and pharmacological properties similar to the Cl^-^-dependent one.

By the way, among multiple sugar metabolic intermediates, including intermediates near PEP in glycolysis, L-2-phosphoglyceric acid, D-2-phosphoglyceric acid, and 3-phosphoglyceric acid were found to be inhibitory for the ATP uptake with the ID50 values being, 0.18, 0.18, and 0.45 mM respectively (Figs. 3*F* and S7). Other compounds, glucose 6-phosphate, fructose 6-phosphate, L-lactate, pyruvate, oxaloacetate, citrate and fumarate, malate, succinate, acetyl-CoA and NAD^+^ at 1 mM each were all ineffective.

### ATP uptake is roughly correlated with Cl^-^ uptake

**Figure 4:**
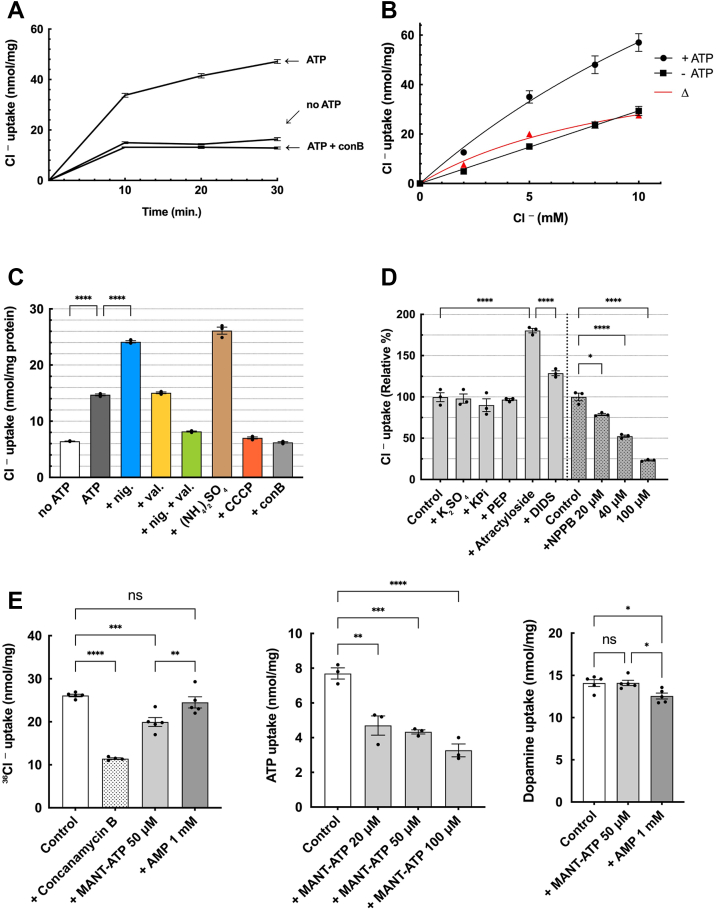
**ATP uptake does not correlate with ^36^Cl^-^ uptake**. *A*, time course of ATP-dependent and -independent ^36^Cl^-^ uptake by CHRMVs ^36^Cl^-^ uptake by CHRMVs was assessed in the *EXPERIMENTAL PROCEDURES* in the presence or absence of ATP. In some experiments, con B at 1 μg was also included in the assay mixture. An aliquot of the assay mixture was taken and filtrated at the incubation time. n = 3. Data represent means ± SEM. Statistical analysis was performed using Student’s *t* test. *B*, Dose dependence of ATP-dependent or independent ^36^Cl^-^ uptake ^36^Cl^-^ uptake by CHRMVs was assessed as described in the presence or absence of ATP at the indicated Cl^-^ concentrations. The difference between the uptake in the presence and absence of ATP at 10 min was also plotted as ATP-dependent. n = 3. Data represent means ± SEM. Statistical analysis was performed using Student’s *t* test. *C*, energetics of ATP-dependent ^36^Cl^-^ uptake ^36^Cl^-^ uptake by K-acetate-trapped CHRMVs was assessed according to the *EXPERIMENTAL PROCEDURES* except that 1 mM ^36^Cl^-^ in the absence or presence of val and nig 0.1 μg each, CCCP 1 μM, con B 1 μM and (NH_4_)2SO_4_ 5 mM. ∗∗∗∗*p* < 0.0001 n = 3. Data represent means ± SEM. Statistical analysis was performed using Student’s *t* test. *D*, pharmacology of ATP-dependent ^36^Cl^-^ uptake (*left*) Con B-sensitive ATP-dependent ^36^Cl^-^ uptake by CHRMVs at 40 min was assessed as described in the absence or presence of the listed compounds: K_2_SO_4_ and KPi at 5 mM each, 1 mM PEP, 50 μM atractyloside, and 2 μM DIDS. 100% activity corresponded to 20.5 nmol/mg protein for at 40 min ∗∗∗∗*p* < 0.0001 (*right*) Con *B*-sensitive ATP-dependent ^36^Cl^-^ uptake by CHRMVs at 50 min was also shown in the absence (control) and presence of NPPB at the indicated concentrations. 100% activity corresponded to 24.0 nmol/mg protein for at 50 min n = 3. Data represent means ± SEM. Statistical analysis was performed using Student’s *t* test. ∗*p* = 0.0336, ∗∗∗∗*p* < 0.0001 *E*. The effect of nucleotides on ATP-dependent uptakes of Cl^-^, ATP and dopamine. *left* ATP-dependent Cl^-^ uptake measured according to the *EXPERIMENTAL PROCEDURES* in the presence or absence (control) of MANT-ATP and AMP at the indicated concentrations. n = 3 Data represent means ± SEM. ∗∗*p* < 0.01, ∗∗∗*p* < 0.001, ∗∗∗∗*p* < 0.0001, ns = not significant. *center* ATP-dependent, conB-sensitive ATP uptake was measured according to the *EXPERMENTAL PROCEDURES* and the legend of Figure 1*A* in the presence or absence (control) of MANT-ATP and AMP at the indicated concentrations. The uptake value in the presence of conB was subtracted. n = 3. Data represent means ± SEM. ∗∗ = 0.0014, ∗∗∗*p* < 0.001, ∗∗∗∗*p* < 0.0001, ns = not significant. *right* ATP-dependent, conB-sensitive dopamine uptake measured according to the *EXPERIMENTAL PROCEDURES* and the legend of Figure 1*A* in the presence or absence (control) of MANT-ATP and AMP at the indicated concentrations. The uptake value in the presence of conB was subtracted. n = 5. 100% control corresponded to 24.7 nmol/mg protein at 50 min. Data represent means ± SEM. ∗*p* < 0.05, ns, not significant.

To reveal the mechanism of how Cl^-^ regulates ATP uptake, it would be necessary to determine whether Cl^-^ is taken up by CHRMVs during ATP uptake. As shown in Figure 4*A* and Figure S8, ATP evoked ^36^Cl^-^ uptake by CHRMVs in a time-dependent fashion. In the absence of ATP, the membrane vesicles took up ^36^Cl^-^ dose-dependently in a non-saturable manner. Unlike ATP uptake, ^36^Cl uptake does not require an ATP-regenerating system and saturates relatively quickly. At the same time, ATP-independent uptake increased linearly with Cl^-^ concentration; the addition of ATP enhanced ^36^Cl^-^ uptake. The difference (ATP-dependent uptake) showed a saturation curve concerning Cl^-^ concentration, and kinetic analysis showed Km value being ∼20 mM (Figs. 4*B* and S9). This ATP-dependent Cl^-^ uptake was wholly inhibited by CCCP and con B (Fig. 4*C*) and was promoted by nigericin in the presence of K^+^, while valinomycin had almost no effect - the addition of both severely suppressed uptake. They are promoted by ammonium sulfate 5 mM. These results indicate that Cl^-^ is not only incorporated into CHRMVs in an energy-independent manner but also actively incorporated against a concentration gradient, mainly depending on Δψ formed by V-ATPase.

ClC-3, an anion channel that acts as a Cl^-^/H^+^ exchanger by utilizing the H^+^ gradient generated by V-ATPase, is known to be localized in CHRMVs (56). Therefore, it is important to evaluate the contributions of ClC-3 or VNUT to the ATP-dependent Cl^-^ uptake observed in our experiments. We found that atractyroside and DIDS, which inhibit ATP uptake, slightly increased ATP-dependent Cl^-^ uptake, suggesting occurrence of an interaction with VNUT (Fig. 4*D*). Next, we found that NPPB, an anion channel blocker recognized for inhibiting Cl^-^ conductance through SLC17A1 (36), reduced Cl^-^ uptake, with an ID50 of approximately 40 μM (Fig. 4*D*). We then investigated the effects of nucleotides on ATP-dependent Cl^-^ uptake, as VNUT, V-ATPase, and ClC-3 are all nucleotide-binding proteins (9, 57, 58). Nucleotides regulate the activity of these proteins, but the effects can vary significantly depending on the type of nucleotide. For instance, while AMP has little effect on VNUT and V-ATPase, it strongly inhibits Cl-current in ClC-3, even in the presence of ATP (9, 57, 58, 59). To assess the nucleotide affinity of purified human VNUT (hVNUT), we employed photoaffinity labeling with biotin-ATP. We incubated the *Escherichia coli* F-ATPase complex with biotin-11-ATP and Mg^2+^, followed by UV irradiation. The results indicated that biotin-11-ATP primarily labeled the β subunit, which is integral to the active site of ATP hydrolysis, along with the α subunit (Fig. S10*A*). This labeling process required UV irradiation and was inhibited by the addition of ATP, confirming the validity of the nucleotide labeling method. Subsequently, we examined the effects of MANT-ATP, a fluorescent ATP analogue, and AMP on the labeling of hVNUT. We found that MANT-ATP inhibited biotin-ATP labeling at concentrations between 20 and 50 μM, whereas AMP had minimal impact at 1 mM. ATP at 0.5 mM nearly completely inhibited biotin-11-ATP labeling (Fig. S10, *B* and *C*). These findings suggest that AMP does not significantly affect the transport activity of VNUT, which aligns with previous research (9). Additionally, we found that MANT-ATP serves as a substrate and promotes H^+^ transport without influencing ATP-dependent H^+^ transport, indicating that MANT-ATP does not disrupt the formation of proton motive force for secondary transport systems (Fig. S11). Finally, we examined the effects of MANT-ATP and AMP on the ATP-dependent uptake of Cl^-^, ATP, and dopamine (Fig. 4*E*). Our findings showed that MANT-ATP partially inhibited the uptake of Cl^-^ and ATP, while AMP at 1 mM had a negligible effect (Fig. 4*E*, *left and center*). Neither MANT-ATP nor AMP significantly affected ATP-dependent dopamine uptake (Fig. 4*E*, *right*). Collectively, these results support the notion that the majority of the ATP-dependent Cl^-^ uptake is mediated by VNUT.

### Multiple modes of action of anions on ATP uptake

**Figure 5:**
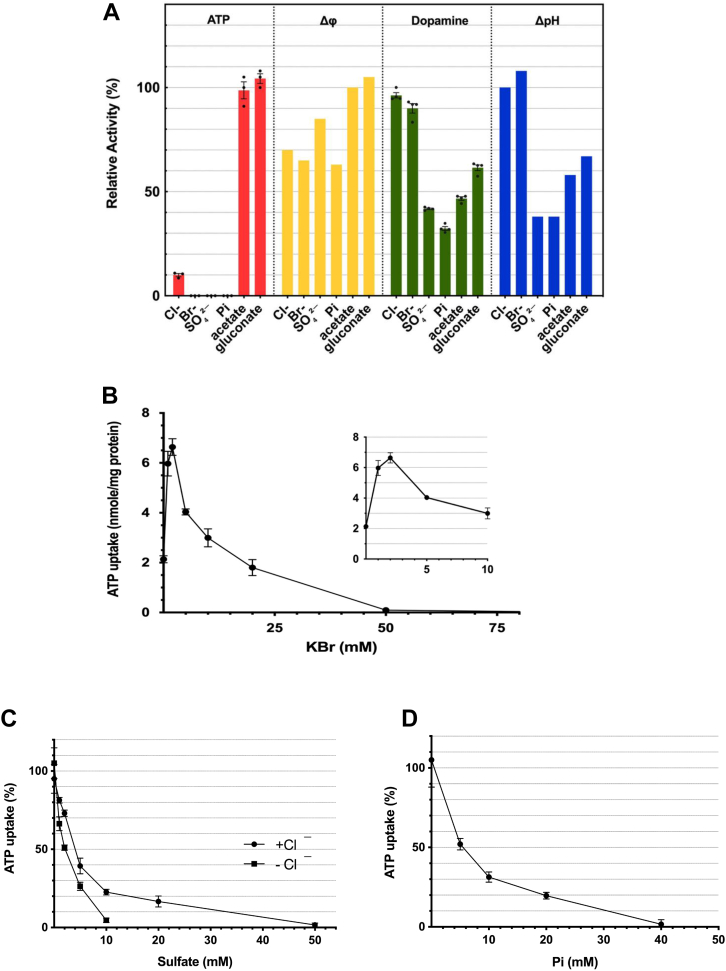
**Classification of anions based on their impact on ATP uptake**. *A*, the effects of various anions at 0.1 M on the ATP-dependent uptakes of ATP and dopamine and the ATP-dependent formation of Δψ and ΔpH were shown. As described in the *EXPERIMENTAL PROCEDURES*, the assay was carried out in the buffer containing either 0.1 M K salts or 50 mM K_2_SO_4_, as indicated. For the ATP uptake, 5 mM KCl was also included in the assay mixture. A 100% activity corresponded to 15.6 nmol/mg protein for ATP uptake, 11.5 nmol/mg protein for dopamine uptake. n = 3. Data represent means ± SEM. Statistical analysis was performed using Student’s *t* test. The ATP-dependent formation of ΔpH and Δψ was estimated according to the legend of Figure 2*A* and shown as a relative value, with the ATP-dependent fluorescence quenching of acridine orange in 0.1 M KCl (45%) and oxonol-V fluorescence quenching (30%) taken as 100%, respectively. n = 1. *B*, dose dependence of Br^-^ on ATP uptake. ATP uptake was assayed under the standard assay condition, except KBr at the indicated concentrations was replaced with KCl. n = 3. Data represent means ± SEM. Statistical analysis was performed using Student’s *t* test. *C*, the dose dependence of SO4^2-^ on ATP uptake in the presence or absence of Cl^-^. According to the *EXPERIMENTAL PROCEDURES*, Cl^-^-dependent and -independent ATP uptake at 40 min was assayed in the presence or absence of SO4^2-^ at the indicated concentrations. Hundred percent activity corresponded to 13 nmol/mg protein for Cl^-^-dependent ATP uptake and 4.1 nmol/mg protein at 40 min for Cl^-^-independent ATP uptake. n = 3. Data represent means ± SEM. Statistical analysis was performed using Student’s *t* test. *D*, the dose dependence of Pi on the Cl^-^-dependent ATP uptake was assayed as described above, except Pi was used instead of SO4^2-^. n = 3. Data represent means ± SEM. Statistical analysis was performed using Student’s *t* test.

To obtain a more comprehensive picture of the effects of anions on ATP uptake, we suspended CHRMVs in a buffer containing 0.1 M of various anions in the form of K salts, and measured the transport of ATP and dopamine, as well as the ATP-dependent formation of Δψ and ΔpH (Fig. 5*A*). We found that ATP uptake was significantly inhibited by Cl^−^, Br^−^, SO_4_^2−^, and Pi, while gluconate and acetate did not. The ATP-dependent formation of Δψ was highest with acetate and gluconate, with about 70% of Δψ being produced by the other anions. Conversely, the formation of ATP-dependent ΔpH exhibited an opposite trend to that of Δψ formation, and dopamine uptake was nearly identical to ΔpH levels, consistent with previous observations (46, 47, 48). These results indicate that ATP uptake is influenced differently by the specific anion species. For instance, Br^-^ had a similar effect to Cl^−^; it promoted ATP uptake at low concentrations but inhibited it at high concentrations, and this effect was observed at lower concentrations than those needed for Cl^−^ (Figs. 5*B* and S12). In contrast, sulfate and Pi did not promote ATP uptake, showing only strong inhibitory effects (Fig. 5, *C* and *D*, Figs S13 and 14). The ID50 for their inhibitory effects was 3 mM, and there was no significant difference in the effects between Cl^−^-dependent and -independent ATP uptake (Fig. 5*C*).

### ATP-dependent sulfate uptake shares properties similar to ATP uptake

**Figure 6:**
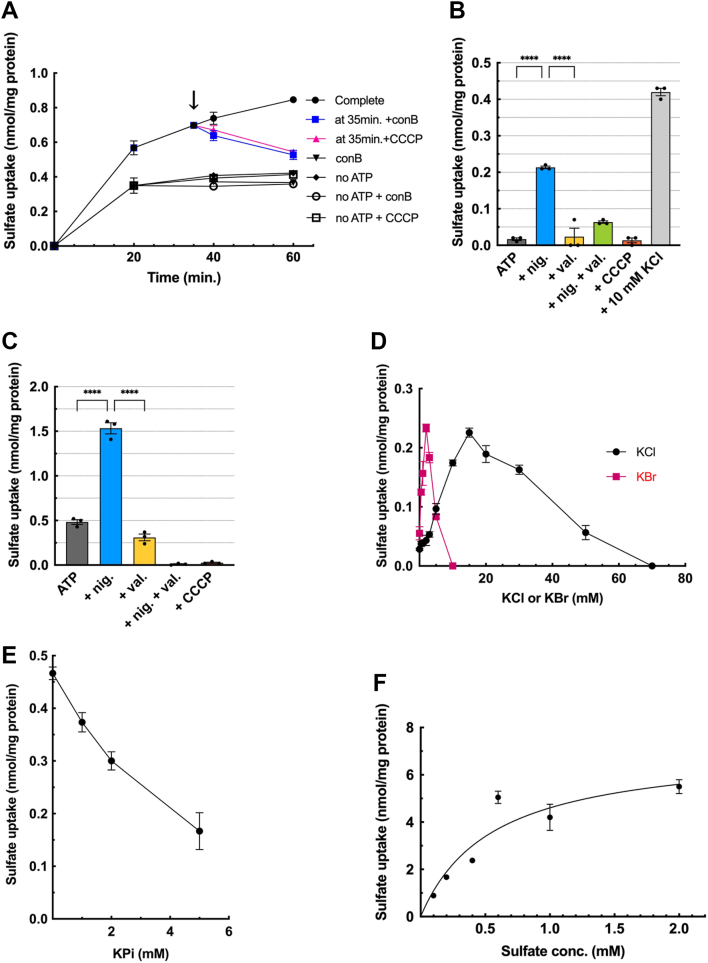

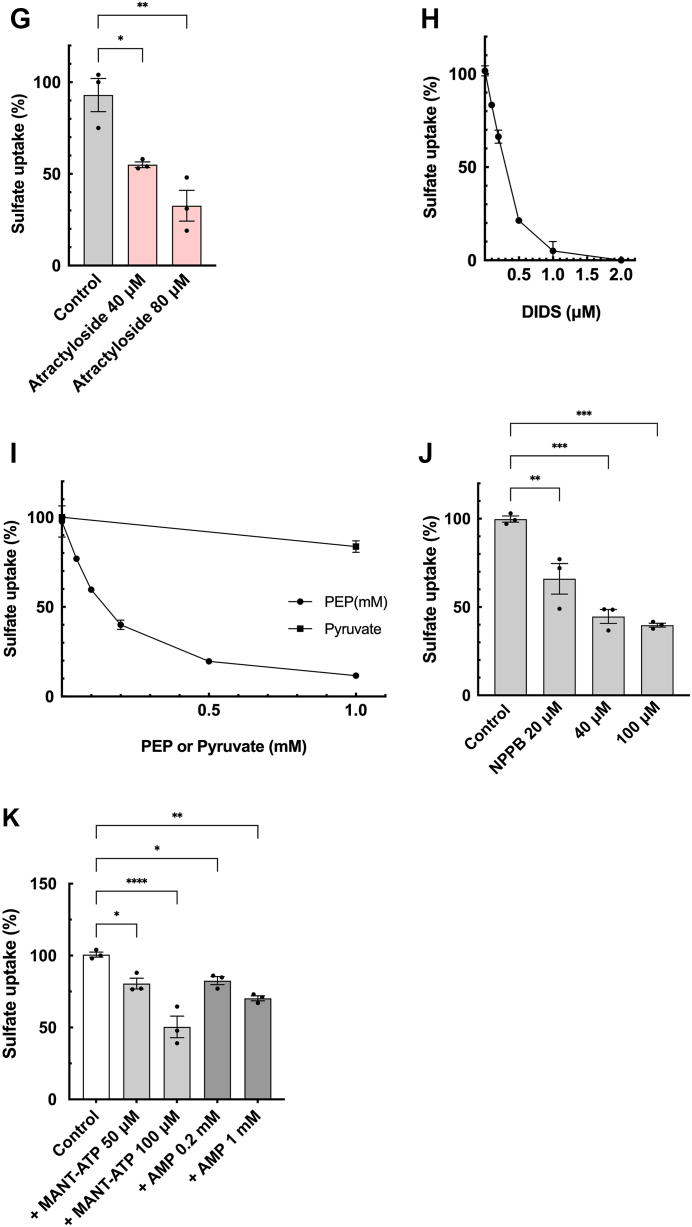
**ATP-dependent [^35^S]SO4^2-^ uptake shares properties of ATP uptake**. *A*, the time course of [^35^S]SO_4_^2-^ uptake by CHRMVs. [^35^S]SO_4_^2-^ uptake was assayed according to the *EXPERIMENTAL PROCEDURES* in the presence or absence of ATP and con *B*. The complete system (●) contains 20 mM MOPS-tris pH 7.0, 0.1 M K-acetate, 10 mM KCl, 0.1 M sucrose, 5 mM Mg-acetate, 1 mM ATP, 0.1 mM [^35^S]SO_4_^2-^ and ATP-regenerating systems. At 30 min incubation, con *B* (■) or CCCP (▲) at 1 μM each were added. The complete plus con B (▼), no ATP (X), no ATP plus con *B* (○), or no ATP plus CCCP (□) were also shown. n = 3. Data represent means ± SEM. Statistical analysis was performed using Student’s *t* test. *B*, ATP-dependent [^35^S]SO4^2-^ uptake by K-acetate-loaded CHRMVs without Cl^-^. Uptake of [^35^S]SO4^2-^ by K-acetate trapped CHRMVs was carried out according to the *EXPERIMENTAL PROCEDURES* in the assay mixture consisting of 20 mM MOPS-tris, pH 7.0, 0.1 M K-acetate, 0.1 M sucrose, 5 mM Mg-acetate, 1 mM ATP, 0.1 mM [^35^S]SO4^2-^ and ATP regenerating systems. ATP-dependent [^35^S]SO4^2-^ uptake at 30 min in the absence of con B was subtracted from those in the presence of con B 1 μM. Additions: none, nigericin (nig) at 0.1 μg, valinomycin (val) at 0.1 μg, CCCP at 1 μM each, 10 mM KCl. n = 3. Data represent means ± SEM. Statistical analysis was performed using Student’s *t* test. ∗∗∗∗*p* < 0.0001. *C*, ATP-dependent [^35^S]SO4^2-^ uptake by K-acetate-loaded CHRMVs in the presence of 10 mM KCl. ATP-dependent [^35^S]SO4^2-^ uptake at 25 min in the absence of con B was subtracted from those in the presence of con B 1 μM. Additions: none, nigericin (nig) at 0.1 μg, valinomycin (val) at 0.1 μg, CCCP at 1 μM each. n = 3. Data represent means ± SEM. Statistical analysis was performed using Student’s *t* test. ∗∗∗∗*p* < 0.0001. *D*, KCl- or KBr-dose dependence on ATP-dependent [^35^S]SO4^2-^uptake. ATP-dependent [^35^S]SO4^2-^ uptake by CHRMVs was carried out under the standard assay condition, except for the concentrations of KCl (●) or KBr (■). n = 3. Data represent means ± SEM. Statistical analysis was performed using Student’s *t* test. *E*, effect of other anions on the ATP-dependent [^35^S]SO4^2-^ uptake. ATP-dependent [^35^S]SO4^2-^ uptake by CHRMVs was carried out as described above except for the concentrations of KPi (●) and K-gluconate (■). n = 3. Data represent means ± SEM. Statistical analysis was performed using Student’s *t* test. *F*, kinetics of ATP-dependent [^35^S]SO4^2-^ uptake [^35^S]SO4^2—^uptake by CHRMVs was assessed in the presence or absence of ATP at the indicated sulfate concentrations. The difference between the uptake in the presence and absence of ATP at 20 min was also plotted as ATP-dependent ones. n = 3. Data represent means ± SEM. Statistical analysis was performed using Student’s *t* test. *G-K*, pharmacology of ATP-dependent [^35^S]SO4^2-^uptake Con B-sensitive ATP-dependent [^35^S]SO4^2-^ uptake by CHRMVs was assessed as described in the absence or presence of the listed compounds: atractyloside (*G*), DIDS (*H*), PEP and pyruvate (*I*), NPPB (*J*), MANT-ATP and AMP (*K*). 100% activity corresponded to 0.6 nmol/mg protein (*G*–*I*), 0.5 nmol/mg protein (*J*), and 0.48 nmol/mg protein (*K*), respectively. n = 3. Data represent means ± SEM. Statistical analysis was performed using Student’s *t* test. In G, ∗*p* = 0.0226, ∗∗*p* = 0.0025. In K, ∗*p* < 0.05, ∗∗*p* < 0.01, ∗∗∗∗*p* < 0.0001.

Based on the effects of anions discussed in the previous section, we hypothesized that VNUT might be capable of transporting sulfate and Pi. To investigate this possibility, we measured the ATP-dependent uptake of [^35^S]sulfate in CHRMVs. As shown in Figure 6*A* and Figure S15, CHRMVs took up [^35^S]sulfate in both ATP-dependent and ATP-independent manner. The ATP-dependent uptake was sensitive to con B and CCCP, while the ATP-independent uptake showed no sensitivity to these compounds. Additionally, after incubating the vesicles with ATP for 30 min, the addition of either con B or CCCP resulted in a decrease in the amount of [^35^S]sulfate taken up, indicating that the [^35^S]sulfate retained within the vesicles was released when the proton motive force was dissipated. These findings suggest that the ATP-dependent uptake of [^35^S]sulfate by CHRMVs is a result of active transport. Conversely, the ATP-independent uptake likely occurs due to non-specific binding to the vesicular membranes.

We measured the ATP-dependent uptake of [^35^S]sulfate by CHRMVs trapped with K-acetate, both in the presence and in the absence of Cl^-^. Without Cl^-^, the ATP-dependent uptake of [^35^S]sulfate was very low. However, this uptake was significantly stimulated by nigericin but strongly inhibited by the addition of either valinomycin or CCCP (Fig. 6*B*). When Cl^-^ was present at a concentration of 10 mM, it promoted ATP-dependent [^35^S]sulfate, which was also significantly enhanced by nigericin and inhibited by either valinomycin or CCCP (Fig. 6, *B* and *C*). These results indicate that Cl^-^ is not essential for ATP-dependent [^35^S]sulfate uptake.

Then, we characterized the influence of anions on ATP-dependent [^35^S]sulfate uptake. As shown in Figure 6*D* and Figure S16, ATP-dependent [^35^S]sulfate uptake reached a maximum of around 10 to 15 mM Cl^-^, rapidly reduced above 15 mM, and reached zero at approximately 50 mM. Br^-^ significantly promoted ATP-dependent [^35^S]sulfate uptake at around 1 to 5 mM concentrations but reduced it above 5 mM. Acetate and gluconate at a concentration of 0.1 M did not affect ATP-dependent [^35^S]sulfate uptake. In contrast, Pi strongly inhibited sulfate uptake at millimolar concentrations (Figs. 6*E* and S17). The ATP-dependent uptake of [^35^S]sulfate exhibited saturable kinetics with a Km value of approximately 1 mM for sulfate (Figs. 6*F* and S18). ATP-dependent [^35^S]sulfate uptake was reduced by about 68% at 80 μM atractyloside. Furthermore, DIDS, PEP, and NPPB also inhibited [^35^S]sulfate uptake, with ID50 values being 0.2 μM, 0.14 mM, and 40 μM, respectively (Fig. 6, *G* and *H*, *I*, *J*, Figs. S19 and 20). MANT-ATP inhibited ATP-dependent [^35^S]sulfate uptake, while AMP at 1 mM was less effective (Fig. 6*K*). These findings indicate that ATP-dependent [^35^S]sulfate uptake shares properties similar to ATP uptake.

### ATP-dependent Pi uptake shares properties similar to ATP uptake

**Figure 7:**
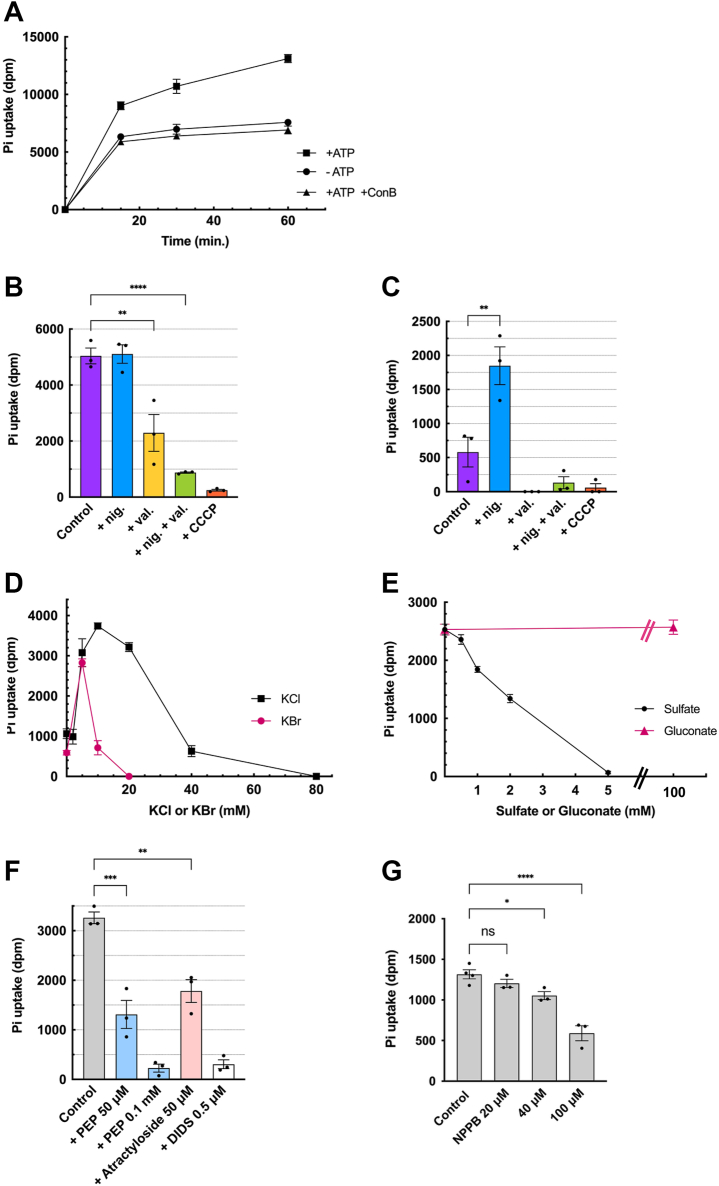
**ATP-dependent [^32^P]Pi uptake shares properties of ATP uptake**. *A*, the time course of [^32^P]Pi uptake by CHRMVs. [^32^P]Pi uptake was assayed according to the *EXPERIMENTAL PROCEDURES* in the presence or absence of ATP and con *B*. The complete system (●) contains 20 mM MOPS-tris pH 7.0, 0.1 M K-acetate, 10 mM KCl, 0.1 M sucrose, 5 mM Mg-acetate, 1 mM ATP, 0.1 mM [^32^P]Pi. At the time indicated, the samples (200 μl) was taken, filtrated, and washed. Then, the radioactivity remained on the filter was counted. n = 3. Data represent means ± SEM. Statistical analysis was performed using Student’s *t* test. *B*, energetics of ATP-dependent [^32^P]Pi uptake. The ATP-dependent [^32^P]Pi uptake at 20 min was assayed in the absence (control) or presence of nigericin at 0.1 μg, valinomycin at 0.1 μg, CCCP at 1 μM. n = 3. Data represent means ± SEM. Statistical analysis was performed using Student’s *t* test. ∗∗*p* = 0.0020. *C*, KCl- or KBr-dose dependence on ATP-dependent [^32^P]Pi uptake. ATP-dependent [^32^P]Pi uptake by CHRMVs was carried out under the standard assay condition, except for the concentrations of KCl (●) or KBr (■). n = 3. Data represent means ± SEM. Statistical analysis was performed using Student’s *t* test. ∗∗*p* = 0.019, ∗∗∗∗*p* < 0.0001. *D*, ATP-dependent [^32^P]Pi uptake by K-acetate-loaded CHRMVs without Cl^-^. Uptake of [^32^P]Pi by K-acetate trapped CHRMVs was carried out according to the *EXPERIMENTAL PROCEDURES* in the assay mixture consisting of 20 mM MOPS-tris, pH 7.0, 0.1 M K-acetate,0.1 M sucrose, 5 mM Mg-acetate, 1 mM ATP, 0.1 mM [^32^P]Pi. ATP-dependent [^32^P]Pi uptake at 30 min in the absence of con *B* was subtracted from those in the presence of conB 1 μM. Additions: none, nigericin at 0.1 μg, valinomycin at 0.1 μg, CCCP at 1 μM each. n = 3. Data represent means ± SEM. Statistical analysis was performed using Student’s *t* test. *E*, effect of sulfate and gluconate on the ATP-dependent [^32^P]Pi uptake. ATP-dependent [^32^P]Pi uptake by CHRMVs was carried out as described above, except for the concentrations of K2SO4 (●) and K-gluconate (■). n = 3. Data represent means ± SEM. Statistical analysis was performed using Student’s *t* test. *F*, *G*, effect of PEP, atractyloside, DIDS and NPPB on the ATP-dependent [^32^P]Pi uptake. ATP-dependent [^32^P]Pi uptake by CHRMVs was carried out as described above in the presence of the listed compounds. n = 3. Data represent means ± SEM. Statistical analysis was performed using Student’s *t* test. ∗∗*p* = 0.0012, ∗∗∗*p* < 0.0001 in (*F*), and NS, not significant. ∗*p* = 0.0387, ∗∗∗∗*p* < 0.0001 in (*G*).

We further investigated whether CHRMVs uptake Pi in an ATP-dependent manner. As shown in Figure 7*A*, incubating [^32^P]Pi with CHRMVs resulted in a high background signal. The addition of ATP significantly increased the amount of [^32^P]Pi uptake in a manner sensitive to con B and CCCP (Fig. 7, *A* and *B* and Fig. S21).

Next, we measured the ATP-dependent [^32^P]Pi uptake in K-acetate-trapped CHRMVs, both in the presence and in the absence of Cl^-^. The ATP-dependent uptake was notably enhanced by nigericin, while valinomycin strongly inhibited it, as did the combination of both agents (Fig. 7*B*). The inclusion of 10 mM Cl^-^ facilitated [^32^P]Pi uptake, which was less affected by nigericin but severely inhibited by either valinomycin or the combination of both (Fig. 7*C*). We observed a dose-dependent activation of [^32^P]Pi uptake by Cl^-^ and Br^-^, with the maximum effect occurring at concentrations of 10 mM for Cl^-^ and 5 mM for Br^-^. However, at higher concentrations, both Cl^-^ and Br^-^ strongly suppressed Pi uptake (Figs. 7*D* and S22). Additionally, sulfate at millimolar concentrations inhibited ATP-dependent [^32^P]Pi uptake, while acetate and gluconate did not show any inhibitory effect even at concentrations up to 100 mM (Figs. 7*E* and S23). Furthermore, Pi uptake was inhibited by 0.5 μM DIDS, 0.1 mM PEP, 50 μM atractyloside, and 40 μM NPPB (Fig. 7, *F* and *G*). These results suggest that ATP-dependent Pi uptake exhibits a driving force, sensitivity to anions, and pharmacological characteristics similar to those observed in ATP and sulfate uptake.

### The ATP-dependent uptake of sulfate and Pi is not detected in the adrenal membrane fraction prepared from *SLC17A9*^*−/−*^ mice

**Figure 8:**
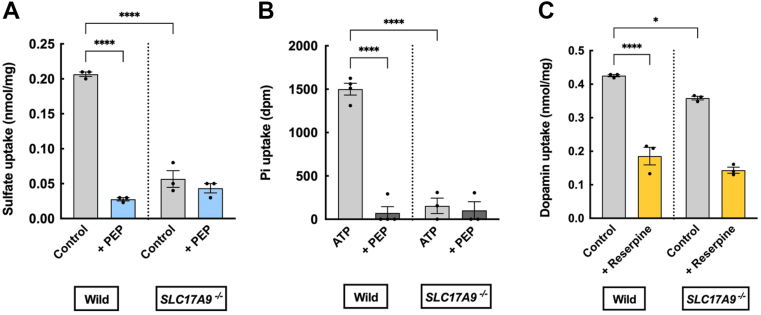
**Loss of ATP-dependent uptakes of [^35^S]SO_4_^2-^ and [^32^P]Pi by the membrane fraction from the adrenal grands of *SLC17A9*^−/−^ mice**. The membrane vesicles were prepared according to the EXPERIMENTAL PROCEDURES, and the ATP-dependent uptakes of [^35^S]SO_4_^2-^ for 30 min (*left*) and [^32^P] Pi for 30 min (*center*) were measured under the standard assay conditions in the presence of 10 mM KCl in the presence or absence of PEP at 1 mM. The values in the presence of con B at 0.5 μM were subtracted. ATP-dependent uptake of [^3^H]dopamine for 30 min in the buffer containing 50 mM KCl in the presence or absence of reserpine at 1 μM (right) was also measured as positive control. ∗*p* = 0.0267, ∗∗∗∗*p* < 0.0001. These results were obtained using membrane fractions (n = 3 or 4). Data represent means ± SEM. Statistical analysis was performed using Student’s *t* test.

To confirm whether VNUT is responsible for the uptake of sulfate and Pi, we prepared membrane fractions from mouse adrenal glands and examined their ability to take up these substances. As shown in Figure 8*A*, the membrane fraction from wild-type mice demonstrated ATP-dependent uptake of [^35^S]sulfate, which was sensitive to PEP. This uptake was absent in the membrane vesicles from *SLC17A9*-knockout (*SLC17A9*^*−/−*^) mice. Similarly, we observed ATP-dependent and PEP-sensitive uptake of Pi in the membrane fractions from wild-type mice, but this activity was not present in the *SLC17A9*^*−/−*^ mice (Fig. 8*B*). In contrast, both membrane vesicle preparations displayed ATP-dependent and reserpine-sensitive uptake of [^3^H]dopamine. The activity measured in the membrane vesicles from *SLC17A9*^*−/−*^ mice was approximately 70% of that observed in the wild-type mice, which aligns with findings from previous reports (Fig. 8*C*) (15, 16).

## Discussion

Chromaffin granules are the first identified and best-characterized organelles for vesicular ATP filling. Their lumen is acidic with a pH of about 5.6 and contains about 0.17 M nucleotides (including ∼ 0.1 M ATP), about 0.5 M catecholamines, and acidic proteins such as chromogranins as major components (1, 2, 6, 8, 11, 47, 48, 60, 61, 62). Early in their research, several groups found that isolated chromaffin granules take up radioactive ATP, suggesting the presence of an ATP transporter (47, 48, 63, 64, 65). Winkler and colleagues reported that the mitochondrial ATP/ADP exchange inhibitor atractyloside, the glycolytic intermediates PEP, Pi, sulfate, and SCN^-^ inhibit ATP uptake (55). Based on these observations, they proposed that the ATP transporter is an anion transporter with low substrate specificity. However, this was before the discovery of V-ATPase, so they measured these uptakes with chromaffin granules in the presence of EDTA, in which V-ATPase does not function. Furthermore, the presence of membrane-impermeable, osmotically inert acidic substances, along with the presence of a high concentration of intraluminal ATP, leads to the spontaneous generation of ΔpH (acidic inside) and Δψ (negative inside) when the granules are immersed in a neutral buffer, complicating the analysis of the energetics for ATP transport (46, 47, 48, 60, 61, 62, 63, 64, 65, 66, 67). Just as membrane vesicles derived from bacterial cells have provided a powerful tool for analyzing the active transport of ions and nutrients (68), it was expected that membrane vesicles obtained by hypotonic treatment of chromaffin granules and the addition of an ATP regeneration system to the assay solution would overcome the abovementioned problems, providing a reliable and straightforward research platform for ATP uptake. However, contrary to expectations, no progress has been made, and it is still in the preliminary stages of drawing a complete picture of ATP uptake. This study aims to overcome the current situation and obtain detailed insight into the mechanism and regulation of vesicular ATP filling. As a first step in this study, we investigated the effect of Cl^-^ on ATP uptake.

## Cl^-^ is not mandatory but a regulatory factor for vesicular ATP filling

We focused the Cl^-^ requirement on ATP uptake by CHRMVs to reveal any differences between the ATP uptake by CHRMVs and VNUT in proteoliposomes (9, 41). CHRMVs utilize the electrochemical proton gradient established by V-ATPase to take up ATP into the vesicles. In contrast, in VNUT-containing proteoliposomes, ATP uptake is driven by the diffusion potential of K^+^ generated by the addition of valinomycin with the K^+^-containing medium. In both systems, the activation of ATP uptake is strongly influenced by the extravesicular Cl^-^ concentrations. We showed that the pattern of activation in CHRMVs is very similar to that of VNUT-containing proteoliposomes. The optimal Cl^-^ concentration for activation is roughly proportional to the ATP concentration in the assay solution (Fig. 2*B inset*): The significant difference between the results reported by Brankston and Guidotti and those obtained with proteoliposomes can be attributed to the ATP concentration in their assay system, which was 50 times higher than that in the proteoliposomes. Although the exact reason for this phenomenon is not yet fully understood; it is possible that ATP interferes with Cl^-^ binding on VNUT or Cl^-^ conductance through VNUT, resulting in a loss of allosteric activation for ATP uptake. Given that cytoplasmic ATP concentrations are estimated to be around 2 to 4.4 mM and Cl^-^ concentrations are estimated to be between 10 and 20 mM, VNUT can maintain nearly complete activity within the cells (69, 70, 71).

Cl^-^ conductance in VGLUTs and SLC17A1 is closely related to Cl^-^-dependent transport activation (29, 30, 31, 36). Since the Cl^-^ dependence of ATP uptake by CHRMVs is similar to the effect observed in synaptic vesicles, synaptic-like microvesicles, and VGLUT-containing proteoliposomes (9, 35, 37, 38, 39, 40, 53), it is reasonable to assume that VNUT-mediated ATP uptake involves a Cl^-^ conductance similar to that of VGLUTs or SLC17A1. To investigate this, we measured ATP-dependent ^36^Cl^-^ uptake by CHRMVs. Our findings demonstrated that CHRMVs take up Cl^-^ in an ATP-dependent manner coupled to V-ATPase. This uptake process differs from ATP uptake in terms of the pharmacological effects of PEP, DIDS, and atractyloside. However, NPPB, an anion channel blocker that inhibits Cl^-^ conductance when SLC17A1 is expressed in *Xenopus oocytes*, also inhibited Cl^-^ uptake in CHRMVs within a similar concentration range. Additionally, the ATP-dependent Cl^-^ uptake is sensitive to MANT-ATP, similar to ATP uptake. Its insensitivity to AMP suggests that ClC-3 is not responsible for this activity. Thus, these findings support the idea that VNUT is responsible for the observed ATP-dependent Cl^-^ uptake; further studies, particularly examining the interaction of MANT-ATP with ClC-3, will be necessary.

There are some differences in Cl^-^ dependence between ATP uptake by CHRMVs and VNUT-containing proteoliposomes. In the proteoliposomes, ATP uptake was rarely observed in the absence of Cl^-^ (9). However, in CHRMVs, comparable ATP uptake occurs even in the absence of Cl^-^, when appreciable amount of Δψ is supplied by the addition of nigericin in the presence of K^+^. The energetics and pharmacology of Cl^-^-independent ATP uptake are nearly identical to those that depend on Cl^-^. Thus, Δψ predominates over Cl^-^ in ATP uptake, indicating that Cl^-^ is not essential for ATP uptake.

The most significant difference in ATP uptake between CHRMVs and VNUT-containing proteoliposomes occurs when the concentration of Cl^-^ exceeds 20 mM. In this context, ATP uptake in CHRMVs is strongly reduced (Fig. 2, *B* and *D*, and 5*A*). This reduction in transport activity can also be observed in ATP-dependent glutamate uptake in synaptic vesicles, synaptic-like microvesicles, and VGLUT2-containing proteoliposomes reconstituted with F-ATPase (32, 33, 34, 35, 53, 54). In contrast, VNUT-containing proteoliposomes show no apparent inhibitory effect (9). A similar phenomenon is observed in proteoliposomes containing NPT1 (SLC17A1), VGLUT2 (SLC17A6), VGLUT3 (SLC17A8), and sialin (SLC17A5) as well as the plant SLC17-type transporter, ascorbate transporter (AtPHT4;4) (72, 73, 74). The properties of this reduction of the activity can be summarized as follows: (I) It occurs when V- or F-ATPase functions as the primary pump, suggesting that H^+^ transport may be involved. (II) The reduced state does not recover even when the acidic conditions established by V-ATPase are altered to Δψ (inside positive) by adding ammonium ions. (III) The sharper reduction is observed when Cl^-^ is substituted with Br^-^. (IV) This phenomenon occurs regardless of the presence or absence of K^+^ or Pi, indicating that these ions are not directly related to the reduced process. The exact mechanism behind this reduction remains unclear; however, the anion channel mode of VNUT may contribute to the observed reduction. It is possible that the increase of luminal [Cl^-^] and [H^+^] at high Cl^-^ concentrations in the assay medium facilitates efflux of luminal Cl^-^ and ATP through VNUT, resulting in the reduction. Considering these results together, it is reasonable to conclude that Cl^-^ acts as a regulator rather than an essential component of ATP uptake. Further analysis using electrophysiological techniques and proteoliposomes is required to investigate the issue of Cl^-^-conductance in VNUT more thoroughly.

## VNUT is a vesicular transporter for sulfate and Pi

Our extensive investigation into the effects of anions on the ATP uptake in CHRMVs has resulted in classifying anions into three distinct groups. Cl^-^ and Br^-^ are composed of the first group, activating and inhibiting ATP uptake dose-dependently. Br^-^ exerts its action at lower concentrations than Cl^-^, indicating a higher affinity of Br^-^ than Cl^-^ for its binding site(s) of VNUT. Sulfate and Pi do not activate ATP uptake but strongly inhibit it, constituting second group. This inhibitory effect is more pronounced than previously reported and differs from the inhibition caused by high Cl^-^ and Br^-^ concentrations. Acetate and gluconate do not significantly affect ATP uptake and are thus classified in the third group.

We hypothesized that sulfate is an alternative substrate for VNUT. In fact, Winkler and his colleagues, and Phillips and Allison, independently, reported that chromaffin granules incorporate sulfate in a proton-conductor-sensitive fashion (55, 65). In the present study, we demonstrated that sulfate is a transport substrate for VNUT. The energetics of this uptake and the pharmacological effects on the inhibitor agreed with the properties of VNUT.

In mammals, sulfate is the fourth most abundant anion in plasma, ∼300 μM (75). Sulfate originating from food plays an essential role in the formation of many endogenous compounds, such as proteoglycans, steroids, and catecholamines, and the detoxication of many exogenous compounds, such as acetaminophen, isoproterenol and salicylic acid) *via* the sulfate donor 3′-phosphoadenosine-5-phosphosulfate. Because sulfate is a highly dissociated divalent hydrophilic anion, it cannot freely cross the phospholipid bilayer of cell membranes. Thus, transporters are responsible for the influx and efflux into cells and organelles, such as mitochondria. Multiple transporters are involved in sulfate absorption from the gastrointestinal tract, renal tubular reabsorption, and excretion from the cerebrospinal fluid. Because none of the known sulfate transporters are Δψ-driven and have similar pharmacological effects and anion and nucleotide sensitivity, VNUT may be a new category of sulfate transporter.

Since the concentration of free sulfate in the lumen of chromaffin granules seems to be insignificant, sulfate transport by VNUT may be artificial and have no physiological significance. On the other hand, the discovery that VNUT transports sulfate leads to breaking the negative link in energy coupling in ATP transport, in which the consumption of ATP is tightly associated with ATP transport. Thus, VNUT activity in any ATP-filled organelles can be evaluated as ATP-dependent sulfate uptake using radiolabeled sulfate as a substrate.

[^35^S]sulfate has a relatively long half-life of about 2 months, is easy to obtain in high radioactivity, and is an inexpensive and stable substance. Additionally, the membrane orientation of CHRMVs is right-side out, similar to that of Kaback vesicles from *E*. *coli* (64). Consequently, the orientation of VNUT and other transporters within the membrane matches that of intact granules. They can be produced in large quantities, and their ability to transport ATP remains strong even after long-term frozen storage. Moreover, the steps required for measuring VNUT activity are significantly fewer than those needed for VNUT-containing proteoliposomes, resulting in less variability in activity. These properties are advantages that proteoliposomes do not possess. Given these factors, using CHRMVs and sulfate as a substrate offers a straightforward and sensitive method for measuring VNUT activity. This approach is expected to enhance our understanding of VNUT function.

We further found that Pi relatively strongly inhibits both ATP uptake and ATP-dependent sulfate uptake and that, like sulfate, Pi is transported into CHRMVs with energetics and pharmacology similar to ATP uptake, strongly suggesting that Pi is an alternative substrate for VNUT. The discovery that VNUT transports Pi may be important physiologically. A certain amount of Pi exists within the chromaffin granules (61). Although this Pi was considered a degradation product of vesicular nucleotides, the present results raise a possibility that it may originate from cytoplasm. Pi transport driven by VGLUTs has also been shown to be involved in controlling Pi concentration in nerve terminals (29, 76, 77). Thus, similar Pi regulation may also exist in ATP-secreting cells. In any event, it is crucial to analyze sulfate and Pi uptake with VNUT-containing proteoliposomes and Pi dynamics in ATP-secreting cells. These projects are currently ongoing in our laboratory.

## VNUT highlights the new characteristics of the SLC17 family

This study offers new insights into the characteristics of the SLC17 family, which have not been previously explored. As mentioned, VGLUT and VNUT have distinct abilities to recognize substrates, yet their effects on inorganic anions are surprisingly similar. At first, the sensitivity of VGLUT to Cl^-^ is believed to play a crucial role in refilling glutamate and facilitating the recycling process associated with the exocytosis of synaptic vesicles at nerve terminals (29, 30, 31). Therefore, it is possible that Cl-plays a similar regulatory role in the exocytic cycle in ATP-concentrated granules. Second, preliminary findings indicate that VGLUT also transports sulfate in a Δψ-mediated manner, although its activity is considerably lower than that of VNUT (Nomura S *et al*., in preparation). Third, VGLUT has been shown to transport Pi driven by a similar H^+^ electrochemical potential difference. This function is known to help regulate Pi concentration in nerve terminals (29, 76, 77), suggesting that a similar regulatory mechanism for Pi may also exist in ATP-secreting cells. Some comparable functional properties can also be observed in NPT1, a urate exporter (28, 73). Furthermore, sialin (SLC17A5) transports not only sialic acid in a ΔpH-dependent manner but also aspartate in a Δψ-dependent manner (28). Qin *et al*., reported that sialin plays a significant role in the excretion of nitrate (NO_3_^-^) and the bioavailability of nitric oxide (NO) as a ΔpH-dependent NO_3_^-^ transporter when localized to the plasma membrane (78). These findings suggest that all members of the SLC17 family function as transporters for organic and inorganic oxyanions, with Cl^-^ acting as a regulatory element. Understanding the mechanisms behind these properties and their physiological significance remains a vital area for future research.

## Metabolic control of ATP uptake

Finally, this study speculated the presence of an unexpected connection between purinergic chemical transmission and ATP production. Most cancer cells rely on glycolysis to generate ATP, even in an aerobic environment. Still, under blood glucose drops or glycolysis is suppressed, they use ATP from mitochondria through metabolic reprogramming, ensuring survival (79, 80, 81). Since the generated ATP is eventually stored in the secretory vesicles, it is reasonable to believe that vesicular ATP filling and ATP synthesis are metabolically linked. In fact, in hepatocytes cultured under high glucose (25 mM) conditions, the vesicular ATP level increases, which in turn facilitates the rise in the amount of ATP secreted (18, 82). However, it is far less known how vesicular ATP filling is controlled by metabolism.

**Figure 9:**
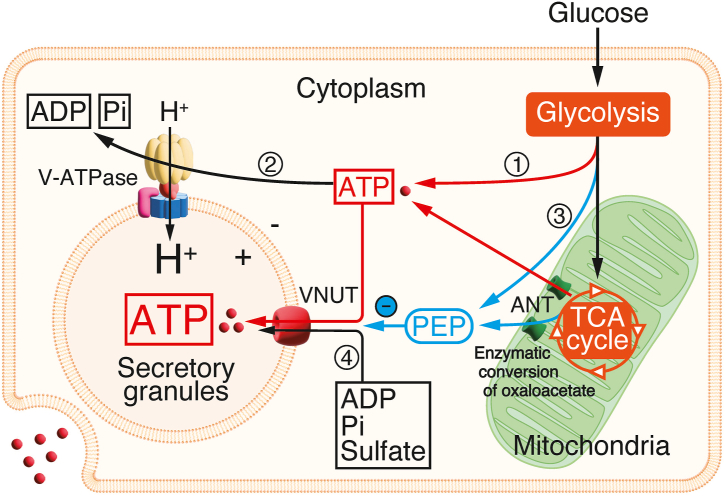
**VNUT as an oxyanion transporter and its potential metabolic control**. (1) ATP produced by glycolysis and oxidative phosphorylation is a substrate for V-ATPase and VNUT (2). V-ATPase pumps H^+^ into the secretory granules at the expense of ATP hydrolysis and establishes proton motive force (ΔpH plus Δψ) across the membrane. Then, VNUT actively transports ATP, using Δψ as a driving force (3). PEP comes from glycolysis and from mitochondria through ATP/ADP exchanger (ANT) by enzymatic oxaloacetate conversion by mitochondrial phosphoenolpyruvate carboxykinase (4). VNUT transports nucleotides, Pi, sulfate, and possibly PEP. PEP inhibits VNUT, causing decreased vesicular ATP filling and release. VNUT activity can be estimated as ATP-dependent sulfate uptake.

PEP, an intermediate glycolysis metabolite with the highest energy phosphate bond in living organisms at −62 kJ/mol, is also synthesized by mitochondrial phosphoenolpyruvate carboxykinase and released from mitochondria through ATP/ADP exchanger (83). As stated above, PEP has been found to inhibit ATP uptake by chromaffin granules at a high concentration of 1 to 10 mM. However, this was previously considered an artificial phenomenon due to the high concentration required for inhibition (55). The present study, however, shows that PEP inhibits V-ATPase-driven uptakes of ATP, sulfate, and Pi to the same extent at physiological concentrations on the sub-mM order. Intermediate metabolites located near PEP during glycolysis also showed similar inhibitory effects, suggesting that PEP plays a key role in regulating vesicular ATP filling as a metabolic regulator under physiological conditions. It is possible that energy production and vesicular ATP filling (purinergic chemical transmission as well) are physiologically linked *via* PEP (Fig. 9). When sugar metabolism becomes active, purinergic chemical transmission is enhanced, but PEP may suppress this activation. Since intracellular concentrations at the mM level can be achieved by adding PEP to the cell culture medium (84, 85, 86), it is also possible to artificially control vesicular ATP filling with PEP *in vivo or in silico*. Adding PEP to the cell culture medium suppresses ATP secretion as the intracellular concentration of PEP increases (Moriyama S *et al*., manuscript in preparation). Further analysis of ATP-dependent ATP uptake inhibition by PEP may lead to developing drugs that suppress purinergic chemical transmission, a potential breakthrough in cancer research.

In this context, the finding that PEP is a negative regulator of Th17 differentiation and functions as a metabolic checkpoint for antitumor T cell responses is particularly fascinating (82, 83). Supplementation of PEP or inhibition of downstream glycolytic enzymes in differentiating Th17 cells increases intracellular PEP levels and inhibits IL-17A expression. PEP supplementation inhibits the expression of characteristic molecules of Th17 and Th2 cells but does not significantly affect glycolysis, cell proliferation, or T helper cell survival. Mechanistically, PEP binds JunB and inhibits the DNA binding of the JunB/essential leucine zipper transcription factor ATF-like (BATF)/interferon regulatory factor 4 (IRF4) complex, thereby regulating the Th17 transcriptional program (87). Daily PEP administration to mice inhibits Th17 cell generation and improves Th17-dependent autoimmune encephalomyelitis. In other words, PEP links aerobic glycolysis and the Th17 transcriptional program, suggesting that PEP could be a promising therapeutic target for autoimmune diseases. The present study further supports vesicular ATP filling as a new target of PEP, sparking further interest in its potential applications.

## In conclusion

This study revealed a detailed picture of the role of the anion in vesicular ATP filling. VNUT is an anion transporter with low substrate specificity that transports nucleotides, Cl^-^, Pi, and sulfate through the Δψ formed by V-ATPase. The Cl^-^ controls the rate of the uptakes as a regulatory factor. PEP at physiological concentrations also regulates the uptakes, suggesting a close metabolic link between purinergic chemical transmission and energy production. The VNUT activity assay utilizing CHRMVs and sulfate ions is a straightforward, sensitive, and user-friendly method to evaluate VNUT function.

## Experimental procedures

### Materials

ATP-tris salt, CCCP, valinomycin, nigericin were obtained from Sigma-Aldrich. Bafilomycin A_1,_ phosphoenolpyruvate, and pyruvate were from Fuji film/Wako. DIDS and atractyloside were from CAYMAN CHEMICAL COMPANY. Concanamycin B (con B) was kindly supplied by Dr Kouichi Ito (Center for Cancer Research, MIT). Creatine kinase from Rabbit muscle (367 units/mg protein) was from ORIENTAL YEAST CO., LTD. Acridine orange hydrochloride and oxonol-V were from TOCRIS and Nihon Kanko Shikiso CO., LTD, respectively. NPPB was from MedChemExpress Co., LTD. Biotin-11-ATP and MANT-ATP were from PerkinElmer and Invitrogen, respectively. ImmunoPure Avidin Horseradish Peroxidase-conjugated was from PIERCE. [2,8-^3^H]adenosine triphosphate tetrasodium salt (26.0 Ci/mmol), 3,4-[RING-2,5,6-^3^H]dihydroxyphenylethylamine (dopamine) (37.8 Ci/mmol), [^32^P] H_3_PO_4_ (2 mCi/ml; 74MBq), [^35^S]Na2SO_4_ (2 mCi/ml; 74 MBq) were obtained from New England Nuclear. [^35^S]Na2SO_4_ (10 mCi/ml; 185 MBq) and [^36^Cl]HCl (0.1 mCi/ml; 16 mCi/gmCl) were obtained from American Radiolabeled Chemicals Inc.

### Preparations

#### CHRMVs

Bovine adrenal glands obtained from a local slaughterhouse were brought to the laboratory in an ice bath. Then, CHRMVs were prepared as described previously with slight modifications (88). All the following procedures were carried out at 4°C or on ice. In brief, adrenal medulla from ∼50 adrenal glands per one preparation (one batch) were suspended in the SME buffer consisting of 20 mM MOPS-tris, pH 7.0, 0.3 M sucrose, 5 mM EDTA, 5 μg/ml pepstatin A, and 5 μg/ml leupeptin. The medullae were homogenized for ∼ 5 seconds in a Waring blender. The mixture was filtered through a nylon filter (Sefar Inc.), and the resultant filtrate was centrifuged at 1000 x g for 15 min. Then, the supernatant was centrifuged at 10,000 x g for 20 min. The pellet was gently suspended in about 30 ml of SME at a 10th of their concentration in the homogenization solution. Then, a fraction (∼10 ml) was applied to the top of the sucrose gradient consisting of 12 ml of 1.8 M sucrose and 15 ml of 1.2 M sucrose, then centrifuged in an SW27 rotor at 20,000 rpm overnight. Then, the solution was removed by aspiration, and the chromaffin granule pellet was collected in a minimal volume of SME. After homogenizing the mixture in a glass-Teflon homogenizer, the suspension was diluted with about 300 ml of the 5 mM MOPS-tris pH 7.0 containing 5 μg/ml pepstatin A and 5 μg/ml leupeptin. After centrifugation at 3000*g* for 10 min, the pellets were discarded, and the supernatant was centrifuged at 200,000×*g* for 1 h. Then, the resultant pellets were homogenized in 5 ml of a solution containing SME and 25%(w/v) glycerol. To prepare KCl or K-acetate-trapped CHRMVs, 10 mM KCl or K-acetate is included in the solution during the hypotonic treatment of chromaffin granules. Additionally, 10 mM of KCl or K-acetate was added to the final suspension of CHRMVs. These membrane vesicles were frozen and kept at −80^o^C until use.

This study utilized 39 batches of CHRMVs and thawed immediately prior to use. ATP, sulfate, Pi, dopamine transport, and V-ATPase activity remained stable and were not affected for up to at least 1 year.

#### Membrane fraction of adrenal glands from wild and *SLC17A9*^*−/−*^ mice

All mouse procedures and protocols were conducted under the Guide for the Care and Use of Laboratory Animals and approved by the Ethics Committee on Animal Experimentation from Kurume University. Membrane fractions of adrenal glands from wild and *SLC17A9*^−/−^ knockout mice were prepared according to the procedure described in ref (16). In brief, fifty adrenal glands isolated from wild and VNUT knockout mice were suspended in SME buffer containing pepstatin A and leupeptin at 10 μg/ml each, homogenized with a Dounce homogenizer 4 strokes, and centrifuged at 1000 x g for 15 min. Then, the supernatant was centrifuged at 10,000 x g for 20 min. The pellet was gently suspended in about 1 ml of SME containing pepstatin A and leupeptin at 10 μg/ml each. Then, the suspension was rapidly poured into 40 ml of 5 mM MOPS-tris pH 7.0 containing 5 μg/ml pepstatin A and 5 μg/ml leupeptin. After centrifugation at 3000 x g for 10 min, the supernatant was centrifuged at 200,000 x g for 1 h. Then, the resultant palettes were homogenized in 5 ml of a solution containing SME and 25%(w/v) glycerol and used for the transport assays. These vesicles were stored at −80°C and were thawed immediately prior to use. We found that ATP, sulfate, Pi, dopamine transport, and V-ATPase activity remained stable and were not affected for up to at least 6 months.

#### Preparation of hVNUT and *E*. *coli* F-ATPase

Procedures for preparation of hVNUT and *E*. *coli* F-ATPase are described in the Supporting Information.

### Assays

#### Uptake of radiolabeled compounds

*[*^*3*^*H]* ***ATP uptake***: In typical experiments, ATP-dependent [^3^H]ATP uptake by CHRMVs was assayed in the buffer 0.5 ml consisting of 20 mM MOPS-tris pH 7.0, 5 mM KCl, 5 m, 0.3 M sucrose, 5 mM Mg acetate, 1 mM [^3^H]ATP (15 KBq/one assay), 0.5 mM creatine phosphate, 10 unit creatine phosphate, and ∼20 μg membrane vesicles as reported previously (89). The ATP concentration, incubation time, and composition of the assay solution were set for each experiment, as shown in the legends. The assay mixture was incubated at 30^o^C, and aliquots (200 μl) were taken at the time intervals and filtered through MF-Millipore 0.45 μm MCE Membrane Filters. Then, the filters were washed with ice-cold 10 ml of 20 mM MOPS-tris pH 7.0, 0.3 M sucrose, 5 mM KCl, 5 mM Mg acetate, and solved in Clear-sol II (Nakalai Tesque). Then, the radioactivity remaining on the filters was counted on a liquid scintillation counter.

#### Uptake of radiolabeled SO4^2-^, Pi, Cl^-^, and dopamine

ATP-dependent [^35^S]SO_4_^2-^uptake was measured in the buffer 0.5 ml consisting of 20 mM MOPS-tris pH 7.0, 0.1 M K-acetate, 10 mM KCl, 5 m, 0.1 M sucrose, 5 mM Mg acetate, 1 mM ATP, 0.1 mM [^35^S]SO_4_^2-^(37kBq/one assay), 0.5 mM creatine phosphate, 10 units creatine phosphate, and ∼10 μg membrane vesicles as otherwise stated (89). In the case of [^32^P] KH_2_PO_4_^2-^ uptake, assay buffer 0.5 ml consisted of 20 mM MOPS-tris pH 7.0, 10 mM KCl, 5 m,0.1 M K-acetate, 0.1 M sucrose, 5 mM Mg acetate, 1 mM ATP, and 0.1 mM [^32^P]KH_2_^32^PO_4_^2-^ (18.5kBq/one assay), and ∼10 μg membrane vesicles were used if otherwise stated.

In the case of ^36^Cl uptake, the assay mixture contains 20 mM MOPS-tris, pH 7.0, 0.3 M sucrose, 5 mM Mg-acetate, 1 mM ATP, 2 mM ^36^Cl^-^ (0.1 μCi/one assay), and ∼10 μg membrane vesicles used. In some experiments, 0.3 M sucrose was replaced with 0.1 M K-acetate and 0.1 M sucrose. In the case with [^3^H] dopamine, in typical experiments, ATP-dependent [^3^H]dopamine uptake was also assayed as described above, except that the buffer consisting of 20 mM MOPS-tris pH 7.0, 0.1 M KCl, 0.1 M sucrose, 5 mM Mg-acetate, 0.1 mM [^3^H]dopamine (10 μM, 15 KBq/one assay) and ∼10 μg membrane vesicles used. Data processing follows that of ATP uptake.

Transport experiments were performed in at least triplicates or more at two different incubation period, indicating n values and are presented as mean ± standard error of the mean (SEM; n = 3–6) as described previously (9, 17, 64). These results were replicated at least twice using different batches of the preparations, providing essentially the same results. Statistical significance was determined by two-tailed paired Student’s *t* test.

#### ATP-dependent H^+^ uptake

Measurement of ATP-dependent formation of ΔpH (acidic inside) and Δψ (inside positive) across the membranes was assayed using fluorescence quenchings of acridine orange and oxonol-V with excitation and emission wavelengths pair being 492 and 540 nm, and 580 and 630 nm, respectively, in 2 ml of the buffer as specified in the legends of figures. Typically, they consist of 20 mM MOPS-tris, pH 7.0, 0.1 K KCl, 0.2 M sucrose, 5 mM Mg acetate, 0.2 μg valinomycin, 2 μM acridine orange, and membranes (∼20 μg for membrane vesicles) for acridine orange, and 20 mM MOPS-tris, pH 7.0, 0.3 M sucrose, 5 mM KCl, 5 mM Mg-acetate, 5 μM oxonol-V and ∼20 μg membrane vesicles for oxonol-V, respectively (89). The degree of ΔpH and Δψ formation due to H^+^ transport was expressed as a percentage of the degree of fluorescence quenching by adding ATP. Typical fluorescence patterns can be seen in ref. 89 and Figure S11. 100% fluorescence intensity corresponds to the intensity before the addition of ATP. In many cases, the addition of ATP causes a slight increase in fluorescence intensity due to interactions with the fluorescent dye, but this value has been corrected. The figure shows the results of single-fluorescence intensity measurements from the relevant experiment. Similar experiments were performed using different batches of preparations, all with comparable values.

### Other procedures

Photoaffinity labeling with biotin-11-ATP, Western blotting, and polyacrylamide gel electrophoresis in the presence of SDS are described in the Supporting information. Protein concentrations were determined using the Bradford method, with bovine serum albumin as a standard, according to the manufacturer's protocol (BIORAD).

## Data availability

Data are available upon request to the corresponding author, Dr Yoshinori MORIYAMA at moriyama_yoshinori@kurume-u.ac.jp.

## Supporting information

This article contains supporting information (90, 91, 92, 93).

## Conflict of interest

The authors declare that they do not have any conflicts of interest with the content of this article.
