# Supplementary material for: Extravesicular chloride ion regulates the uptake of ATP, sulfate, and inorganic phosphate by membrane vesicles from bovine adrenal chromaffin granules
Source: J Biol Chem. 2025 Nov 4;301(12):110892. doi: 10.1016/j.jbc.2025.110892 (PMC12718155; doi:10.1016/j.jbc.2025.110892)
Supplement: Supporting Information [file mmc1.docx]

**Supporting Information**

**Extravesicular Chloride Ion Regulates the Uptake of ATP, Sulfate, and Inorganic**

**Phosphate by Membrane Vesicles from Bovine Adrenal Chromaffin Granules**

Yoshinori MORIYAMA*, Seiji NOMURA, Sawako MORIYAMA, Nao HASUZAWA, and

Masatoshi NOMURA

1

*E. coli* F-ATPase was prepared using a procedure as described previously (2) with a modification. In this modification, the steps of separating the *E. coli* membranes into outer and inner membranes using sucrose density gradient centrifugation and glycerol density gradient centrifugation of the solubilized fraction by octyl-β-glucoside were omitted. The F- ATPase fraction was aliquoted and stored at -80°C, which can be stored stably for at least one year without any loss of activity.

Protein estimation of hVNUT and *E. coli* F-ATPase fraction was performed by the method of Schaffner and Weismann (3).

**SUPPLEMENTAL EXPERIMENTAL PROCEDURES**

**Expression and Purification of Human VNUT (hVNUT) and *E. coli* F-ATPase**

Human VNUT (hVNUT) was expressed and purified as described previously with minor modifications (1). Briefly, *E coli* C43 (DE3) cells were transformed with expression vector pET-β,β hVNUT in TB medium containing 30 μg/ml kanamycin sulfate at 37 °C until the optical density at 600 nm was 0.6 to 0.8. Then, IPTG at a final concentration of 1 mM was added to the medium, followed by incubation for a further 16 h at 18 °C. The following steps were performed at 4°C or on ice. After harvesting the cells by centrifugation, the cells were washed with 20 mM MOPS-Tris, pH. 7.0 containing 0.3 M sucrose and 2 mM PMSF, and suspended in 20 mM MOPS-Tris pH 7.0 containing 0.3 M sucrose, 10 μg/ml leupeptin, and 10 μg/ml pepstatin A. Then the cell suspension was sonicated by Vibra cell^TM^ sonifier (Sonix and Materials Co., Ltd.) with output 25% for 30 sec, 10 times. After removal of debris by centrifugation at 6,000 x g for 10 min, the membrane pellet was obtained by centrifugation at 160,000 x g for one h and suspended in 40 mM Tris-HCl, pH 8.0 containing 0.1 M NaCl, 10 mM KCl, 20%(v/v) glycerol, two mM PMSF, 10 μg/ml leupeptin and 10 μg/ml pepstatin A.

After the protein concentration of the suspension was adjusted to 10 mg/ml, Fos-choline-14 (Anatrace) was added to give a final concentration of 1.5% (w/v). The mixture was then incubated on ice for 10 min, and centrifuged at 160,000 x g for 1 hour. The supernatant was obtained as a solubilized protein fraction. Then, the mixture was applied with Econo-column 1 5 cm x 5 cm (BioRAD) containing 1 ml of Ni-NTA Superflow resin (Quiagen) equilibrated with 20 ml of 40 mM Tris-HCl, pH 8.0, containing 0.1 M NaCl, 10 mM KCl, 20 % (v/v) glycerol, and 20 mM imidazole. After three hours of incubation, the column was successively washed with 40 mM MOPS-Tris, pH 8.0, containing 0.1 M NaCl, 10 mM KCl, 20 %(v/v) glycerol, 20 mM imidazole, and 0.1 % (w/v) *n*-dodecyl-1-thio-β-D-maltoside (DDTM) (Anatrace) and the same buffer containing 50 mM imidazole. Then, the absorbed protein from the resin was eluted with the same buffer containing 0.3 M imidazole. The eluate (purified hVNUT fraction) was divided into small protein fractions and stored at -80 °C until use.

2

**Photoaffinity labeling with biotin-ATP**

Photoaffinity labeling of hVNUT or F-ATPase with biotin-ATP was performed as described previously (4). In brief, purified hVNUT (4 µg) or *E. coli* F-ATPase (10 µg) was suspended in 50 µL of 20 mM MOPS-Tris pH 7.0, 5 mM Mg-acetate, 0.02% (w/v) DDTM, and 10 µM biotin-ATP in the presence or absence of nucleotides. The mixture was kept on ice, and then

illuminated under a UV lamp (254 nm AsOne Handy UV lamp SUV-6 without filter, setting the distance between the UV lamp and the mixture to ~2 cm for 5 min. The labeling reaction was terminated by the addition of 10 µl SDS sample buffer containing 1 % SDS and 10 % β- mercaptoethanol. After ~30 min incubation at room temperature, the sample (3.3 µg for hVNUT or 10 µg for F-ATPase) was subjected to 12.5 % polyacrylamide gel electrophoresis in the presence of SDS. The separated proteins were electrically transferred to nitrocellulose paper and decorated with peroxidase-labeled avidin. Then, biotin-ATP-conjugated hVNUT or F-ATPase was detected by ECL according to the manufacturer’s manual. In parallel with the experiment, the mixture (0.7 µg for hVNUT) was subjected to a 5-20% gradient polyacrylamide gel (ePAGEL E-T520L, Atto Co., Ltd.) in the presence of SDS. After electrophoresis, the gel was

stained with Coomassie brilliant blue to visualize the VNUT protein.

3





**(Fig. 1A)**

**SUPPLEMENTAL FIGURES**

**Fig. S1. Time course of ATP uptake by CHRMVs**

In the original Fig. 1A, many points overlapped, so to make it easier to see, we separated them

into individual time course and listed all points.

4


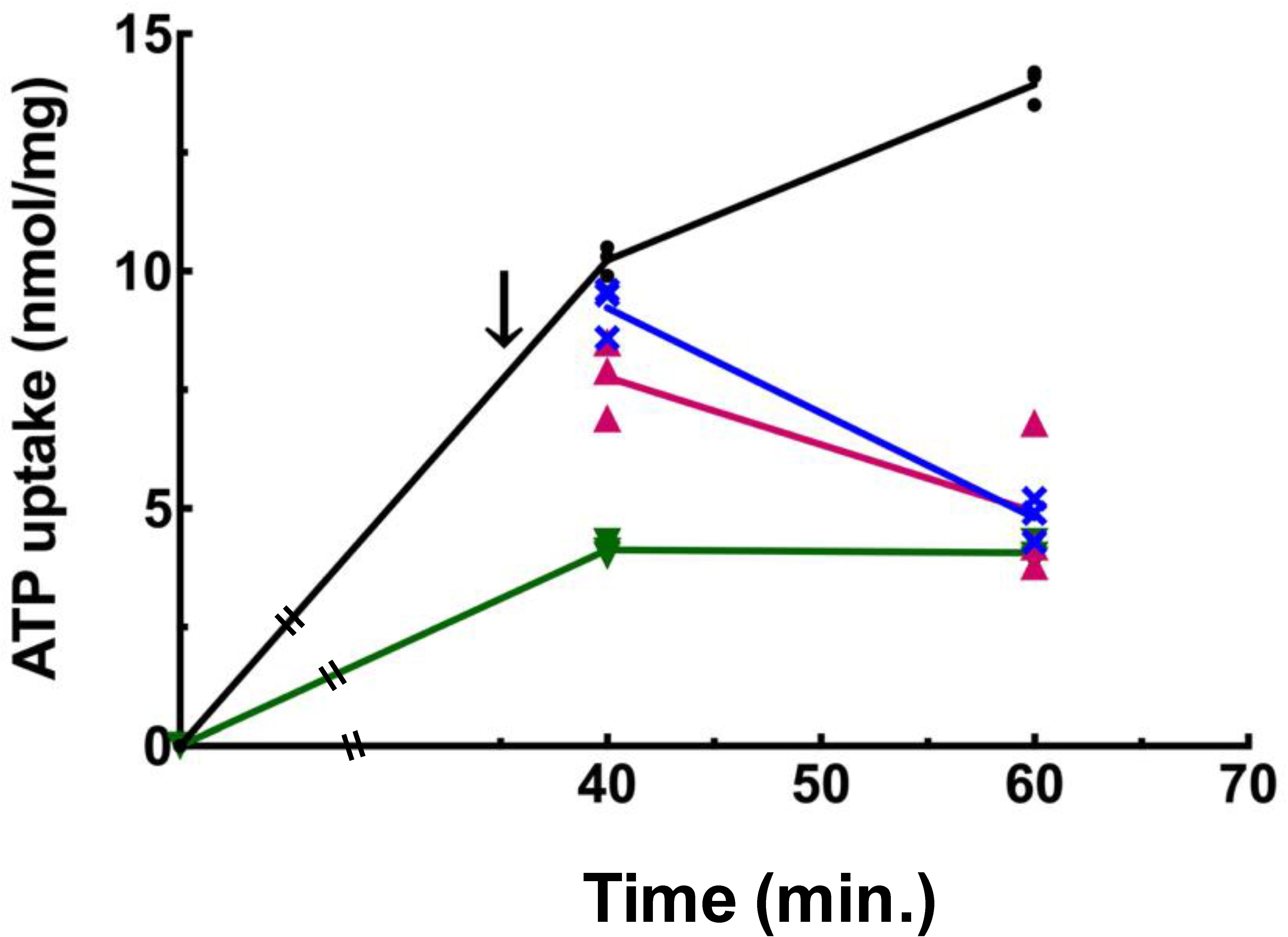


**Fig. S2.**

**All points in Fig. 1B were plotted.**

5


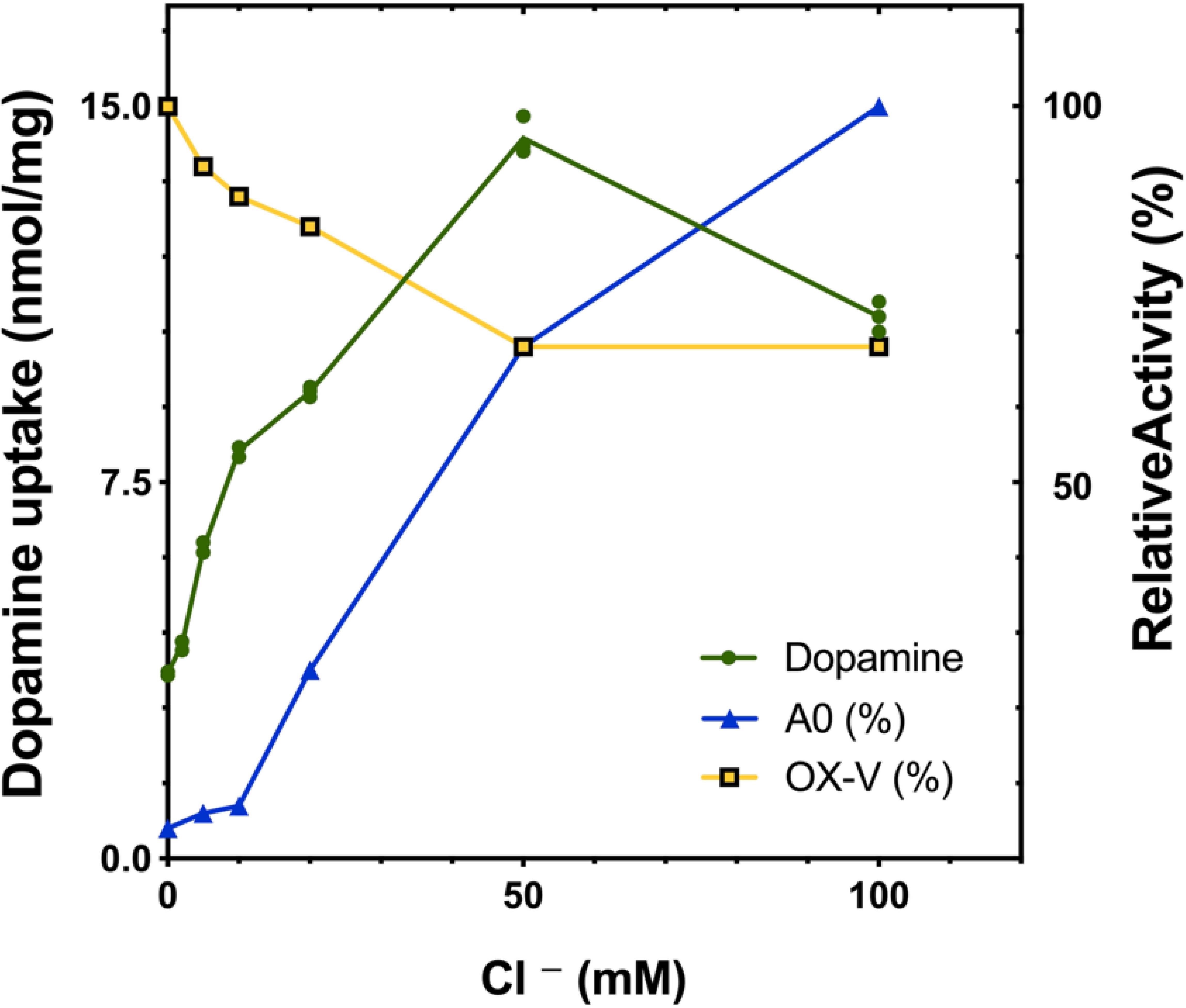

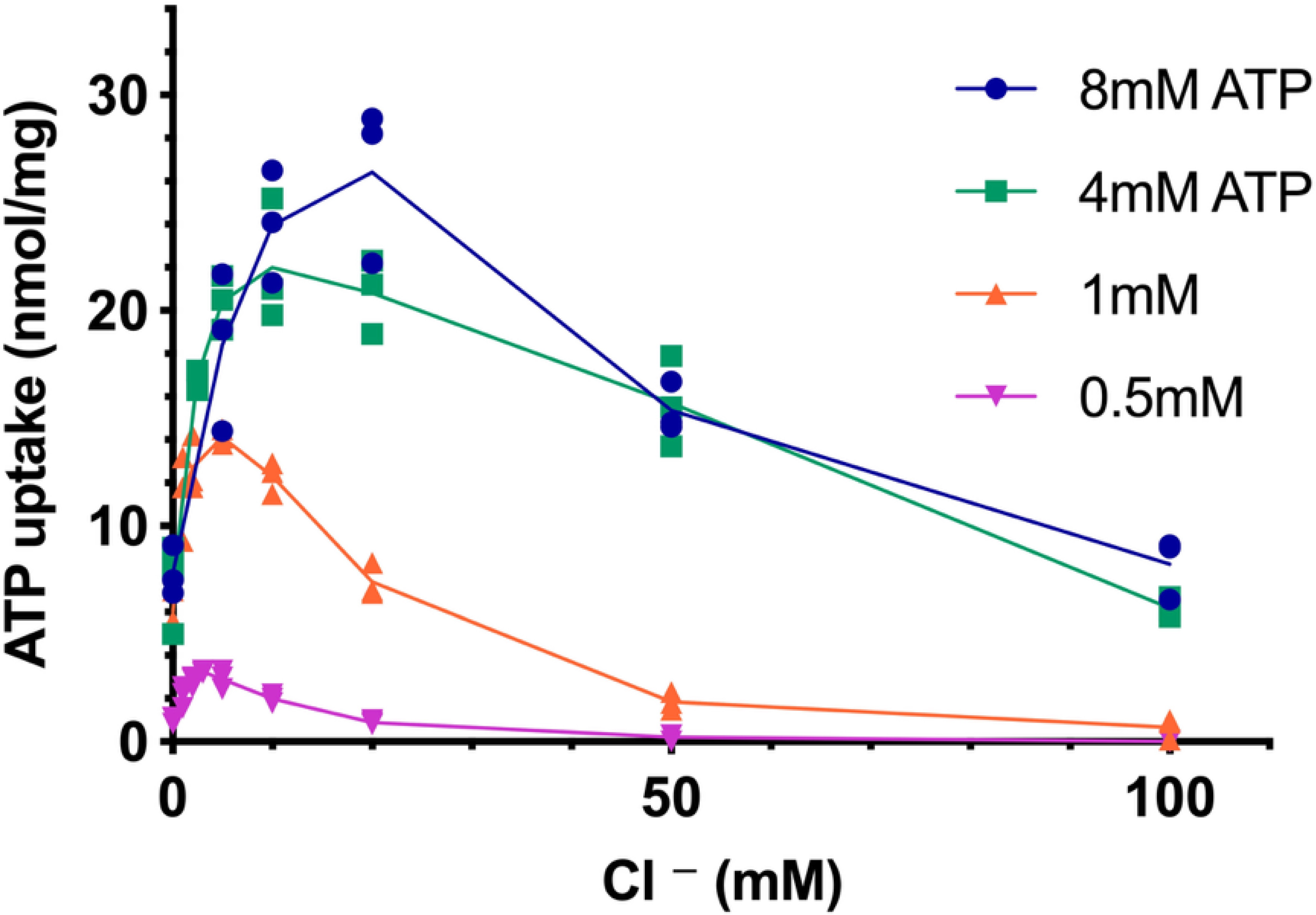


**Fig. S3 and Fig. S4. All points in Fig. 2A and 2B were plotted.**

**Fig.S3**

**Fig.S4**

6


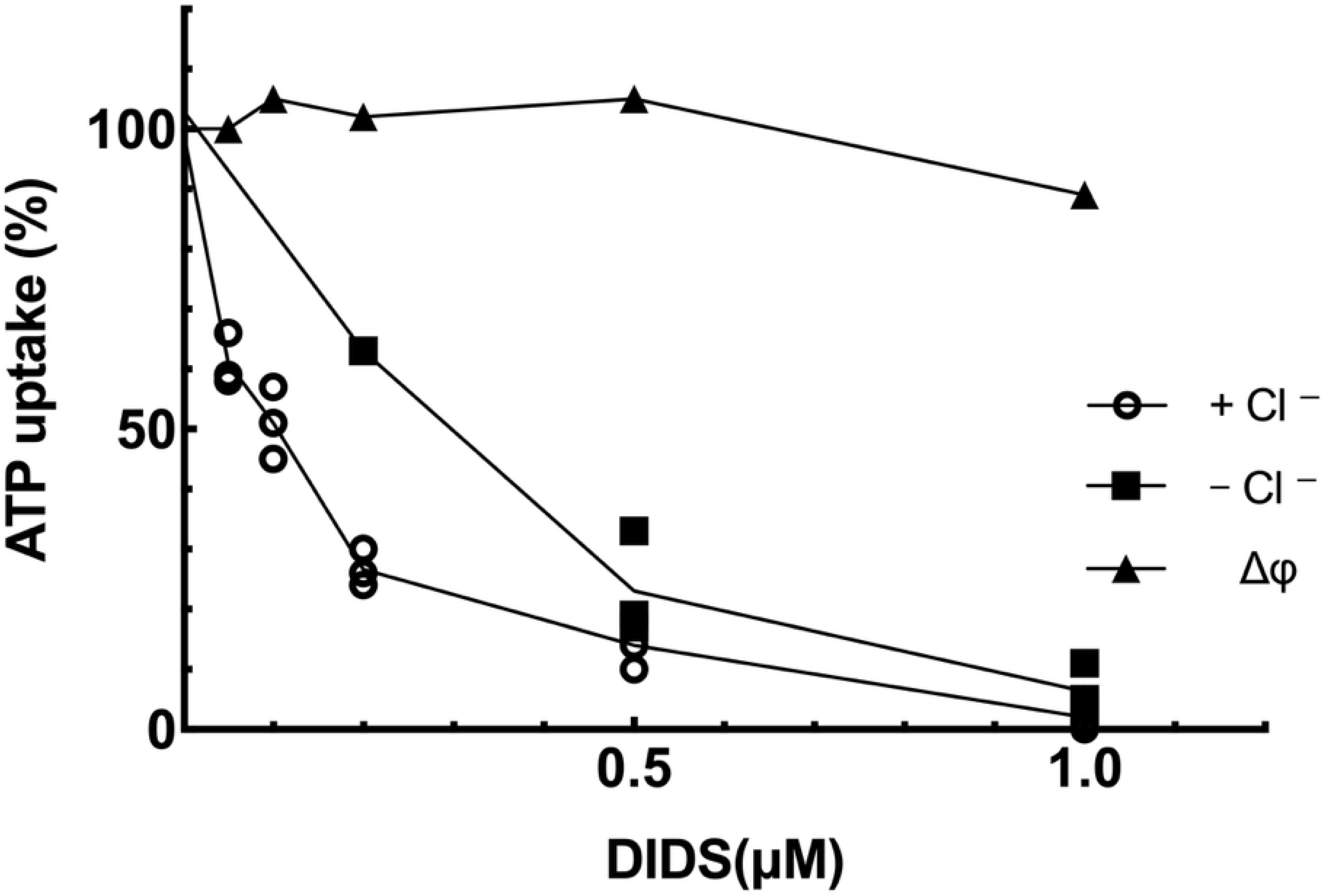

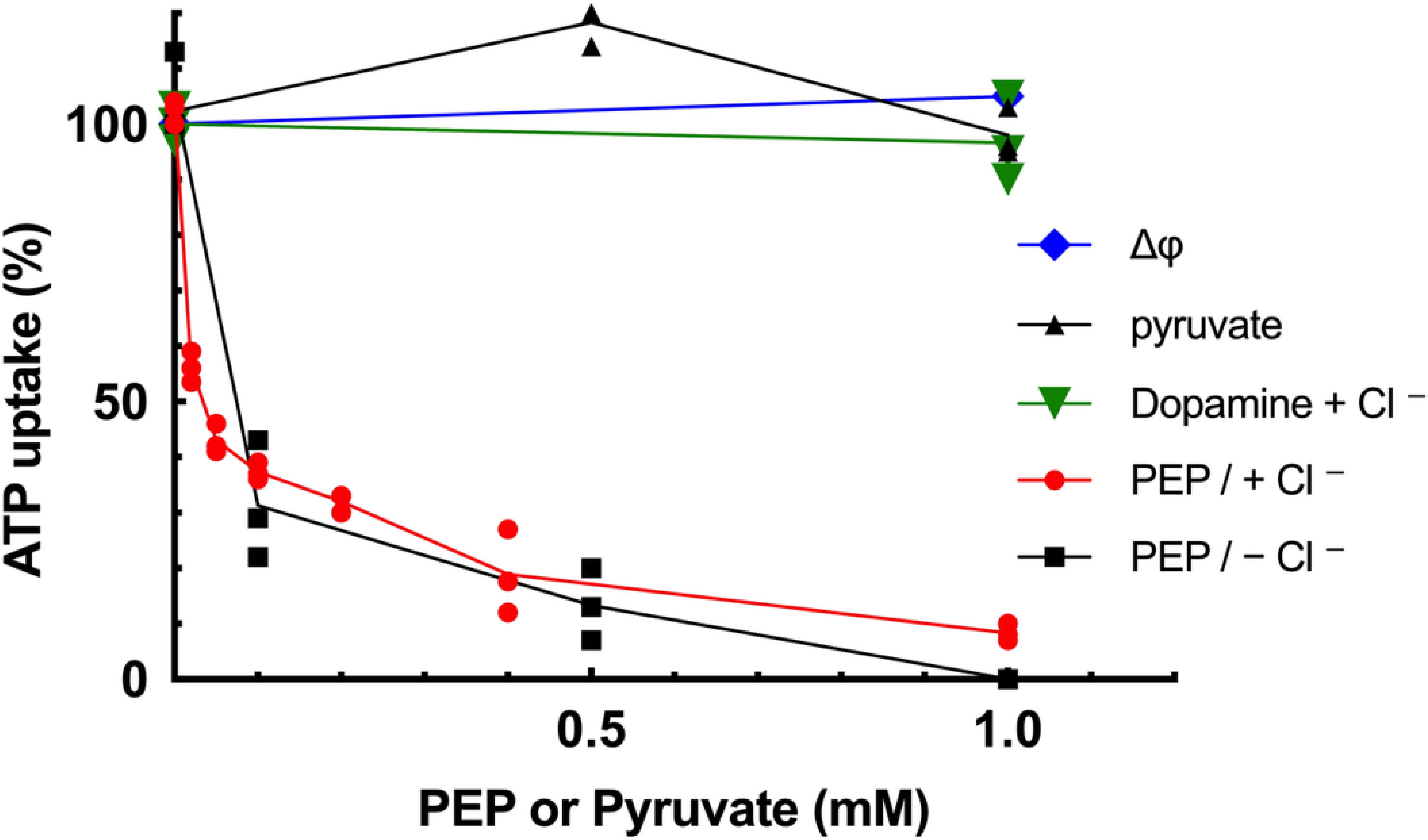


**Fig. S5 and S6 All points in Fig. 3C and 3E were plotted.**

**Fig.S5**

**Fig.S6**

7


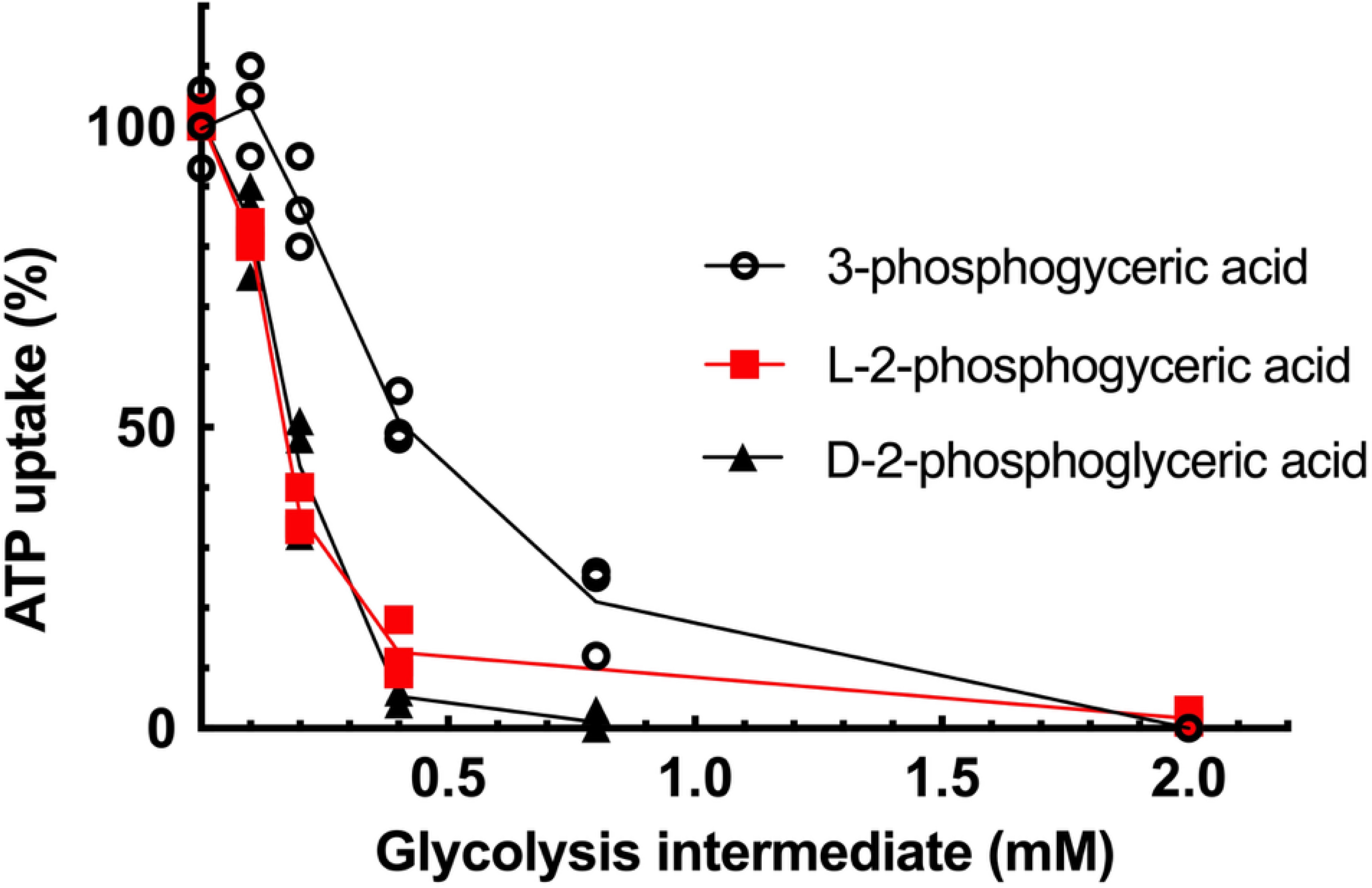


**Fig. S7. All points in Fig. 3F were plotted.**

8


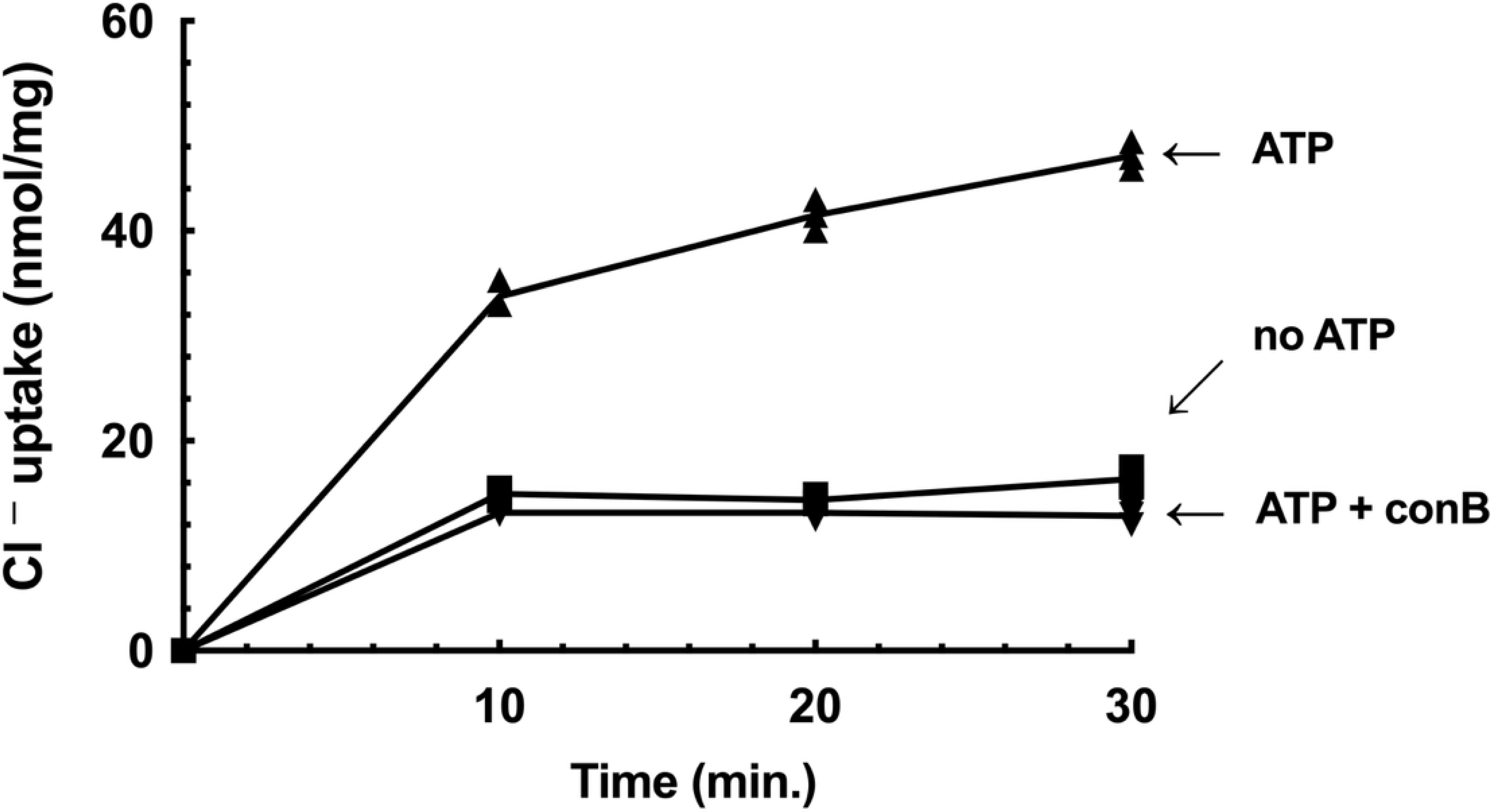

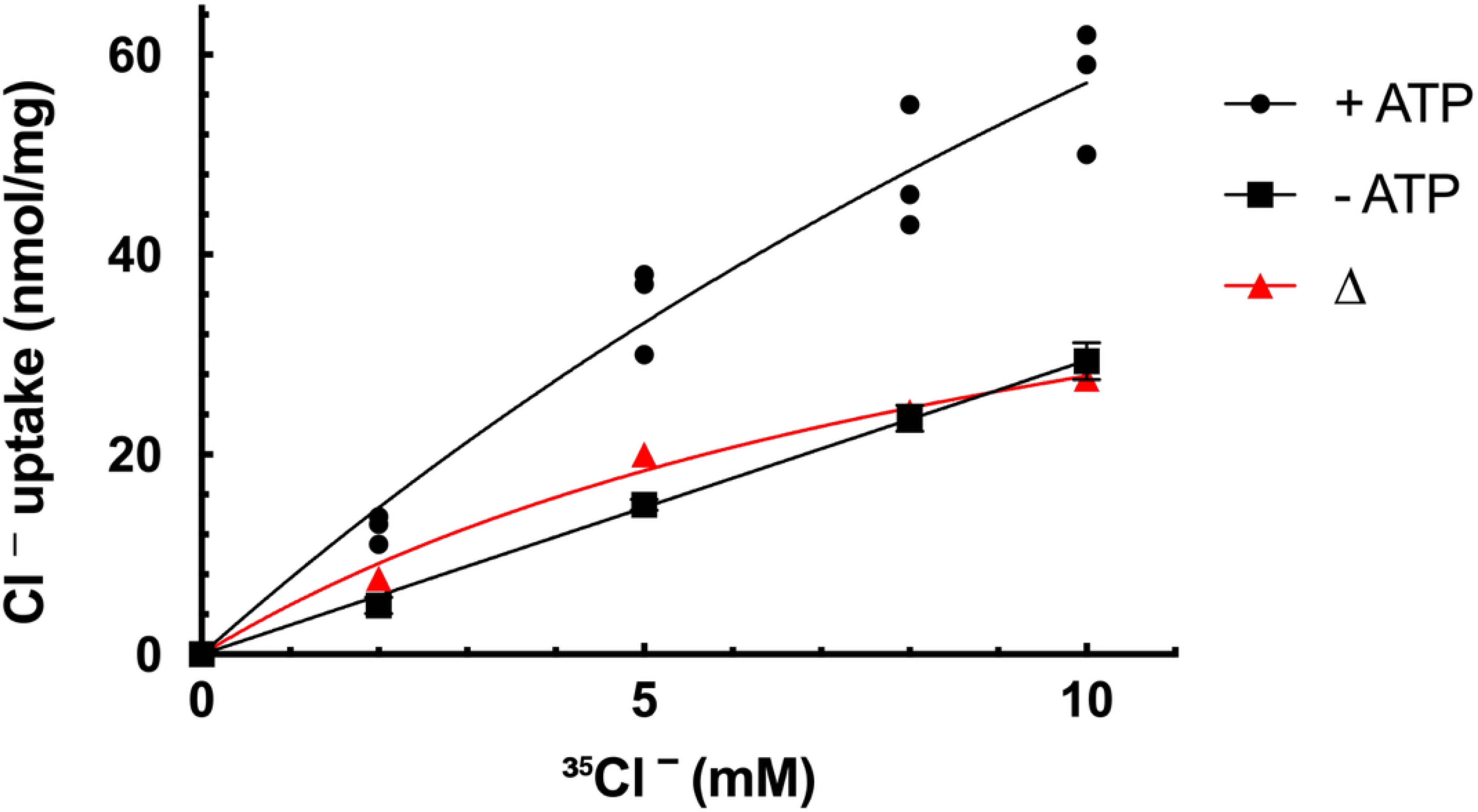


**Fig. S8 and S9. All points in Fig. 4A and 4B were plotted.**

**Fig.S8**

**Fig.S9**

9


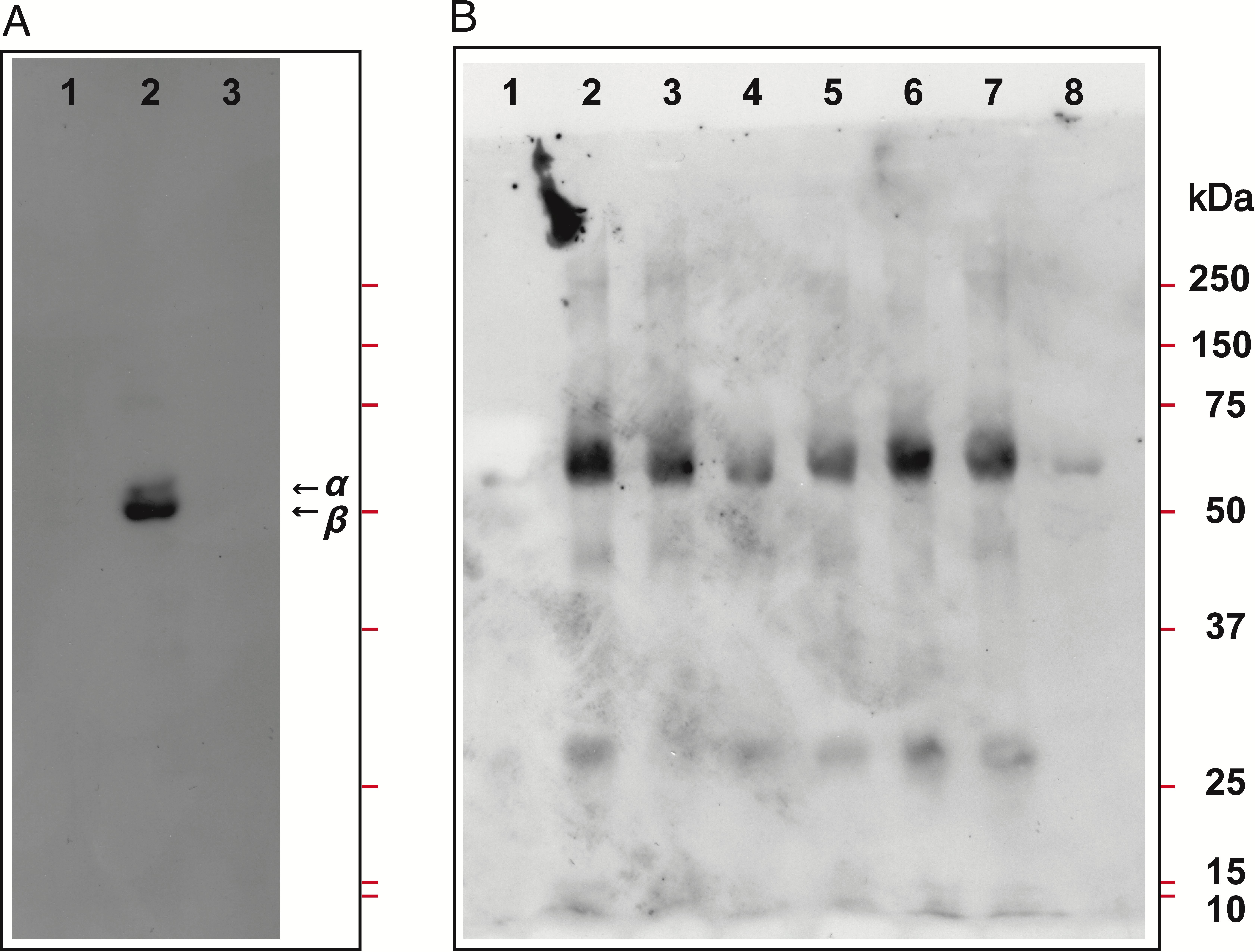


**Fig. S10. Photoaffinity labeling of *E. coli* F-ATPase and hVNUT with biotin-11-ATP.**

1. Biotin-11-ATP labeling with *E. coli* F-ATPase was shown. Lane 1, no UV irradiation control; lane 2, complete system, lane 3, complete system plus 0.5 mM ATP. The position of α, and β subunit was indicated.
2. Biotin-11-ATP labeling of hVNUT was shown. Lane 1, no UV control.; lane 2, complete; lane 3, complete plus MANT-ATP 20 µM, lane 4, complete plus MANT-ATP 50 µM; lane 5 complete plus AMP 0.5 mM; lane 6, complete plus AMP 1 mM; lane 7, complete, line 8, complete plus ATP 0.5 mM. The numbers on the vertical axis indicate the position of the standard proteins using BioRAD precision plus protein^TM^ standard kaleidoscope^TM^.

**C** Coomassie Brilliant Blue staining of F-ATPase (20 µg) and hVNUT (0.7µg) used in A and B.

The position of molecular markers (BioRAD, precision plus protein^TM^ dual color standards) is also indicated.

10


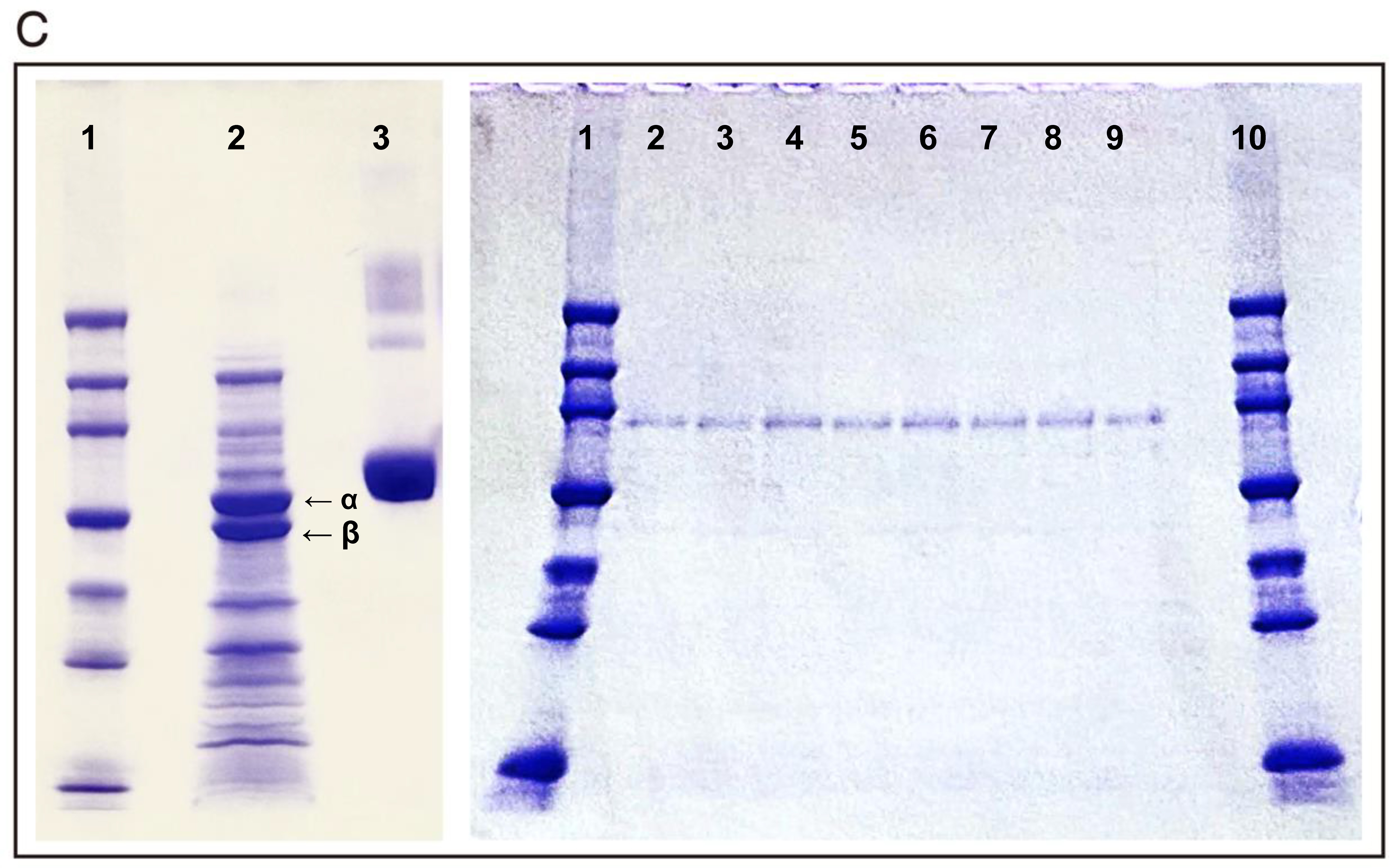


**Fig. S10. (Continued)**

11

**Fig. S11. MANT-ATP-driven formation of Δψ by CHRMVs**

ATP or MANT-ATP-dependent oxonol-V fluorescence quenching were indicated. The numbers on the vertical axis represent the fluorescence intensity (arbitrarily units), and the numbers on the horizontal axis represent the incubation time (min). Additions: ATP 1 mM, MANT-ATP 0.2 mM, and CCCP 0.5 µM.


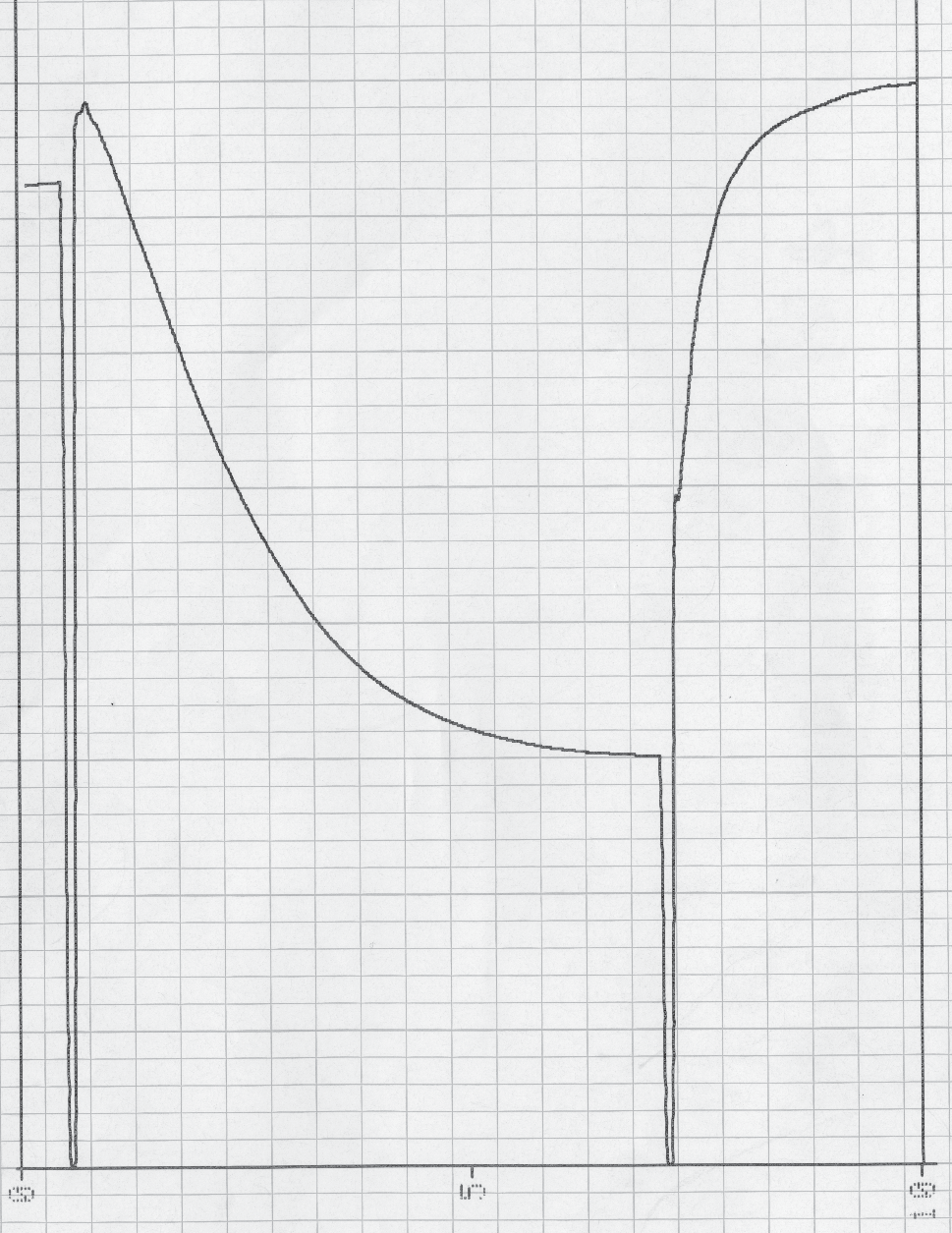

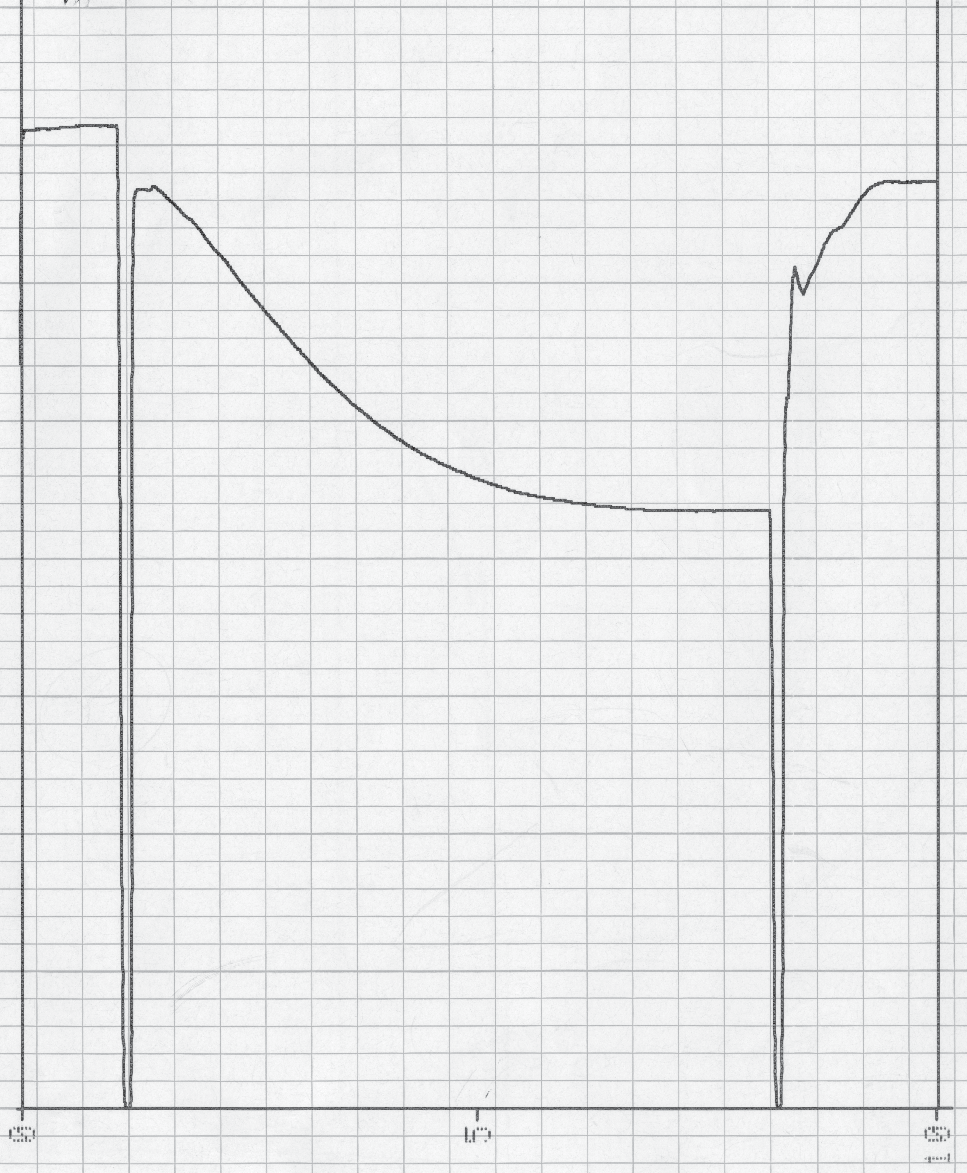


A

B

1400

1400

300

300

Times (min)

Times (min)

12

Relative Fluorescence Intensity (A. U.)

ATP (1 mM)

CCCP (0.5 µM)

Relative Fluorescence Intensity (A. U.)

MANT-ATP (0.2 mM)

CCCP

Relative Fluorescence Intensity (A. U.)

MANT-ATP

ATP

CCCP

C

1400

300

Times (min)

13

**Fig. S11. (Continued)**


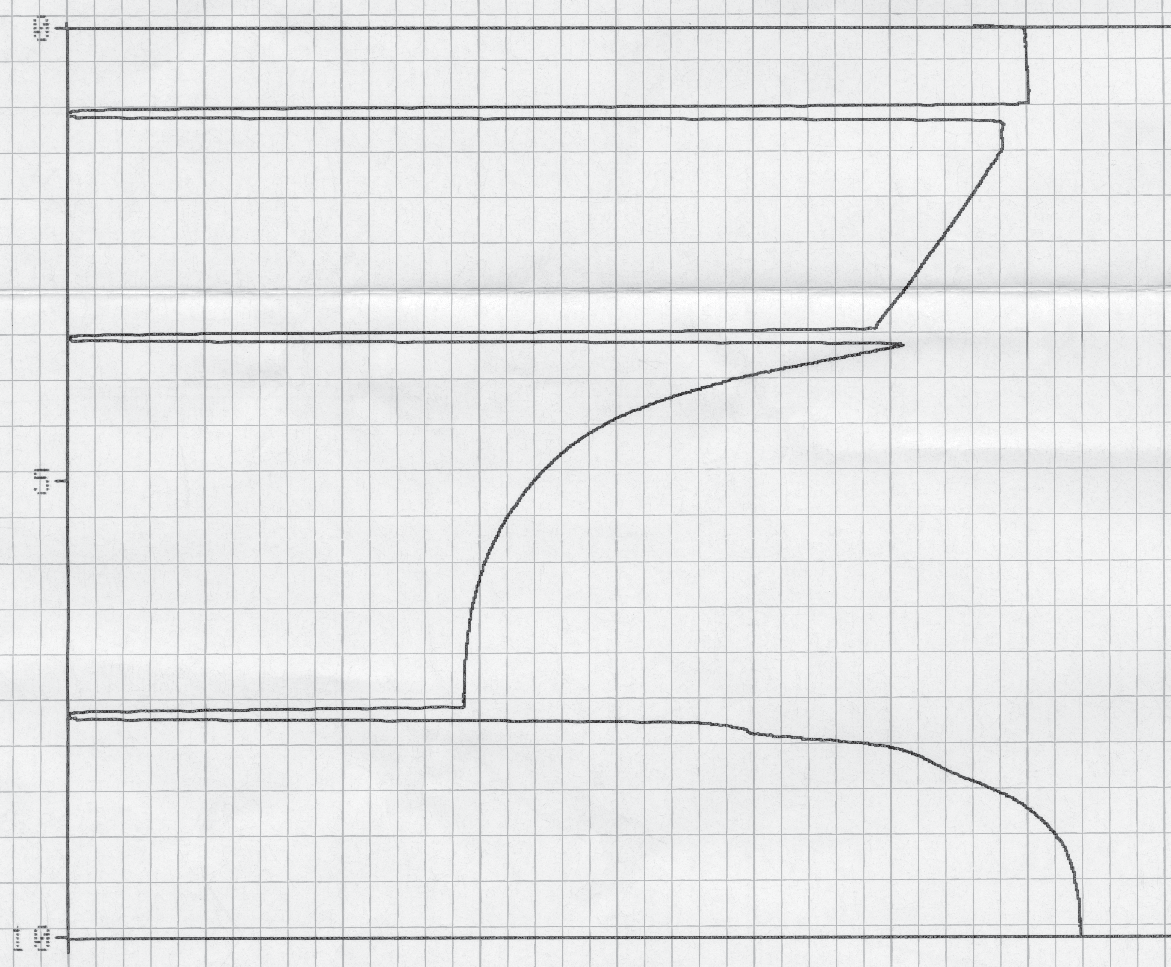


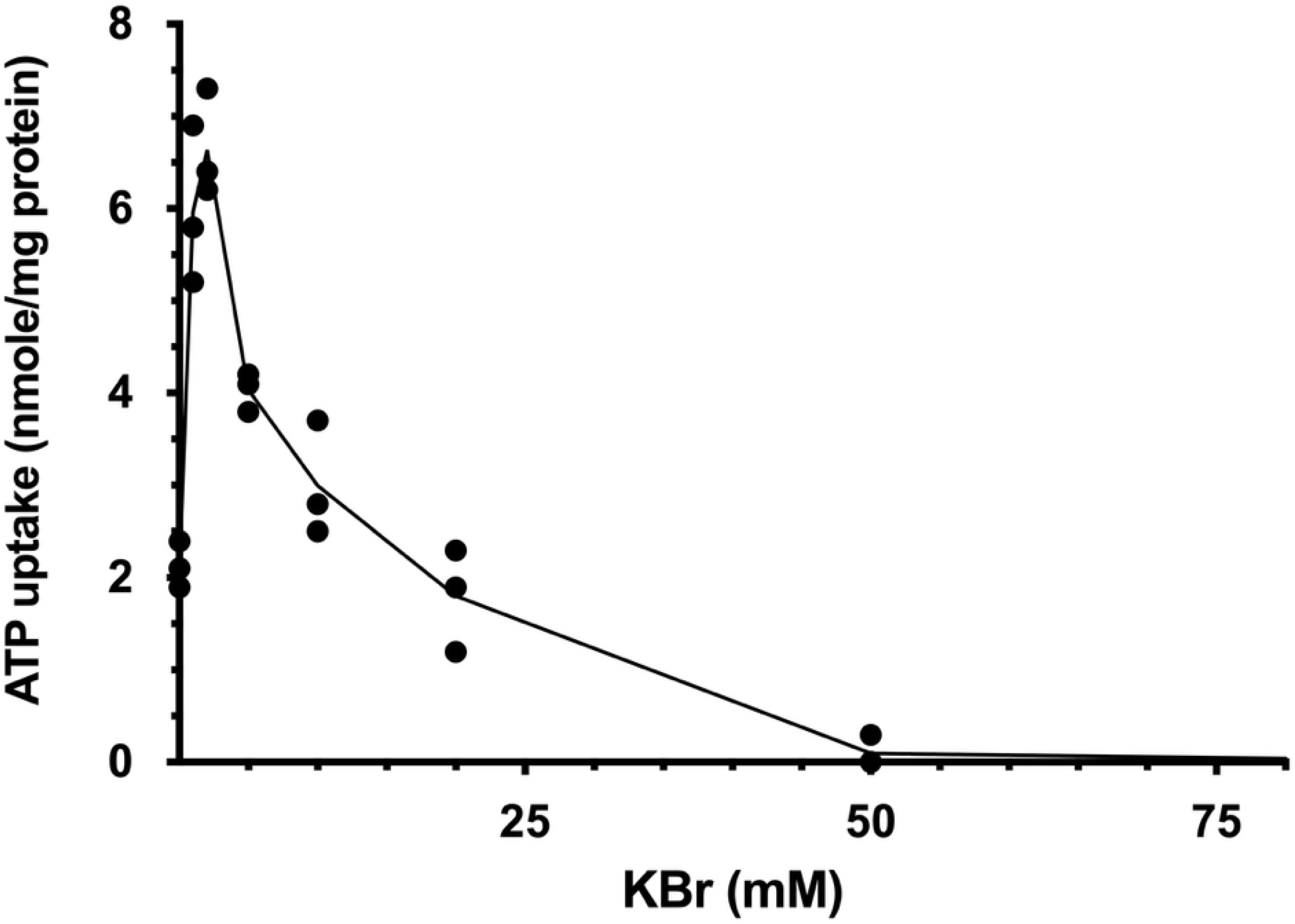


**Fig. S12. All points in Fig. 5B were plotted.**

14


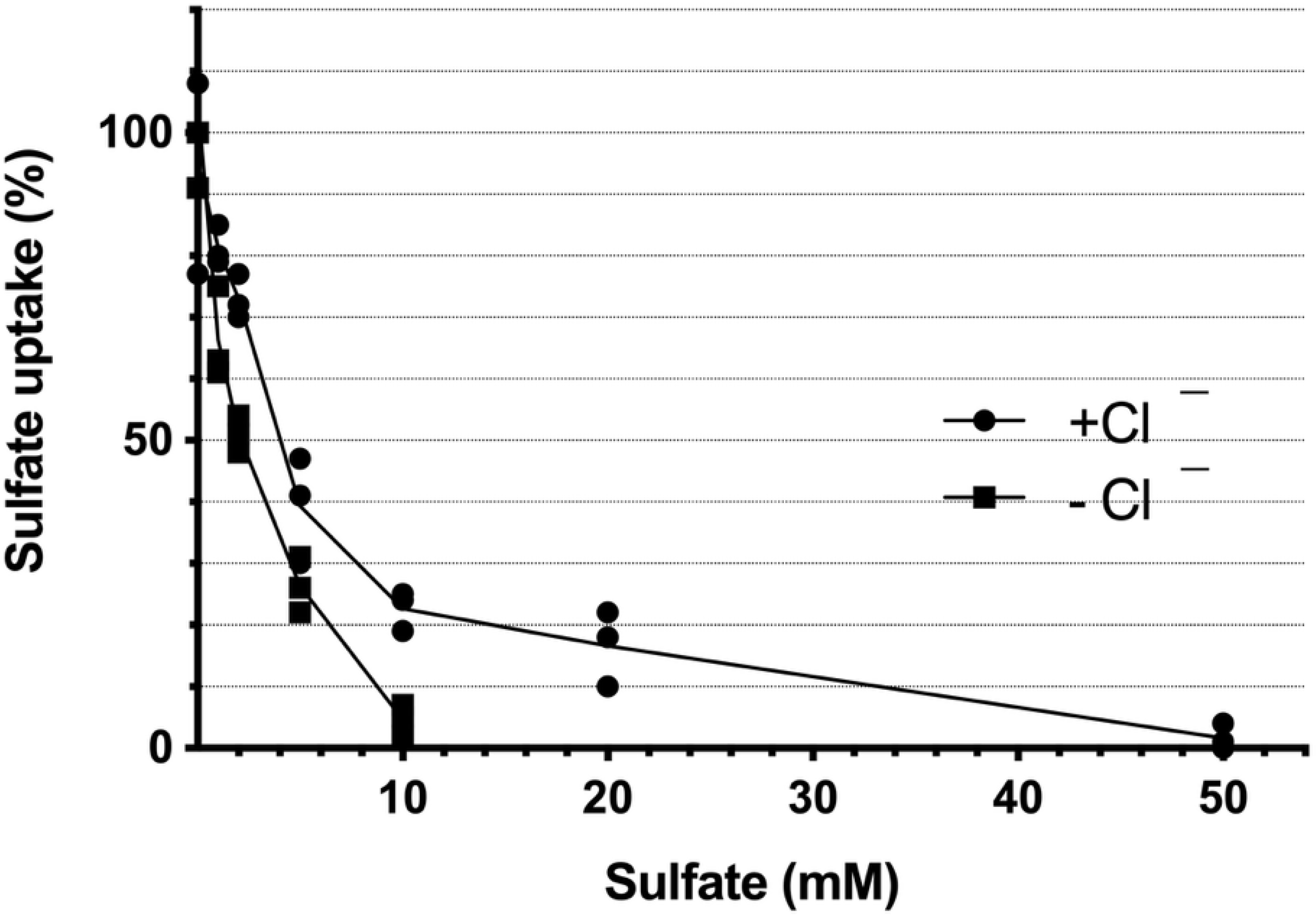

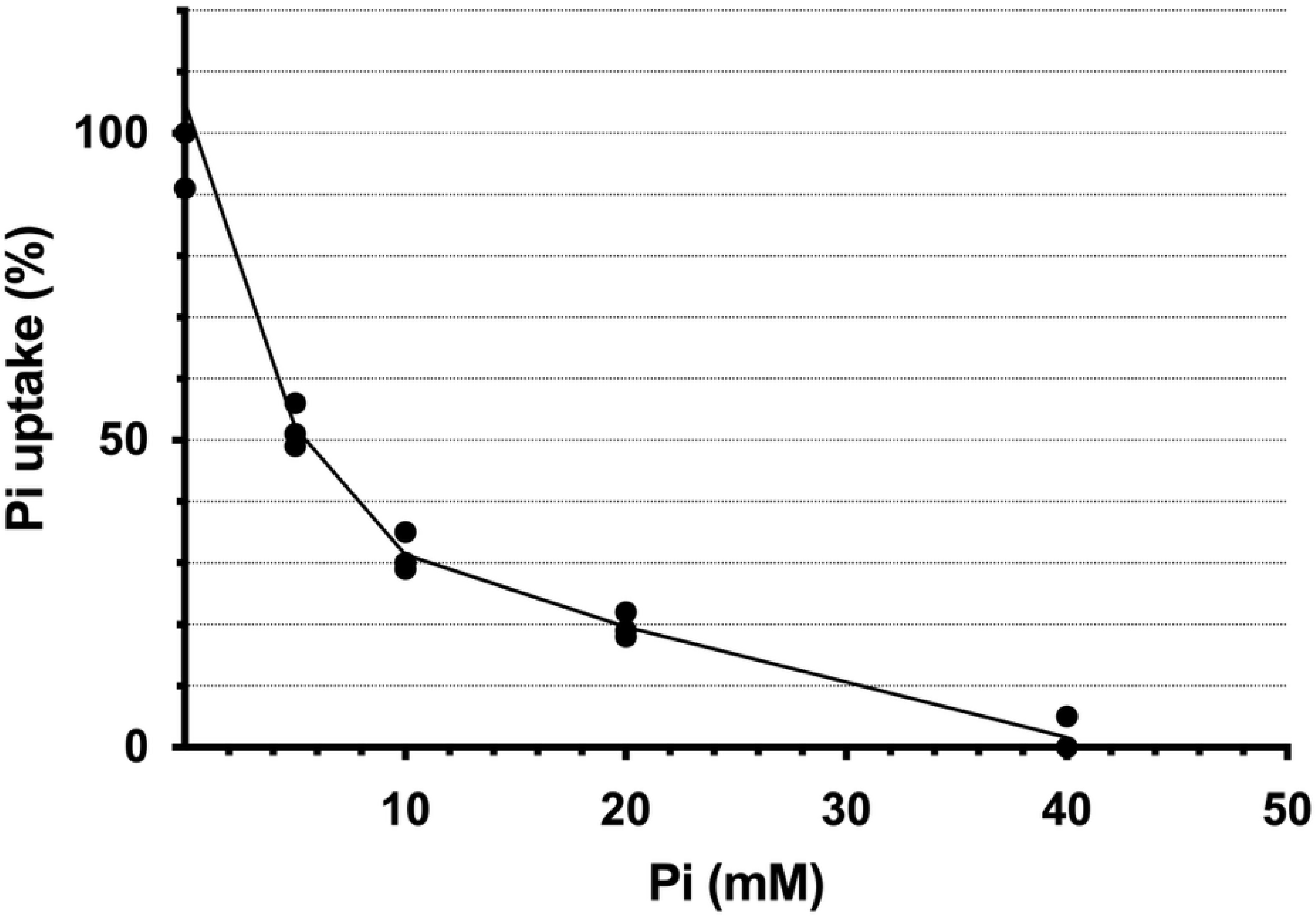


**Fig. S 13 and S14. All points in Figs. 5C and 5D were plotted.**

**Fig.S13**

**Fig.S14**

15


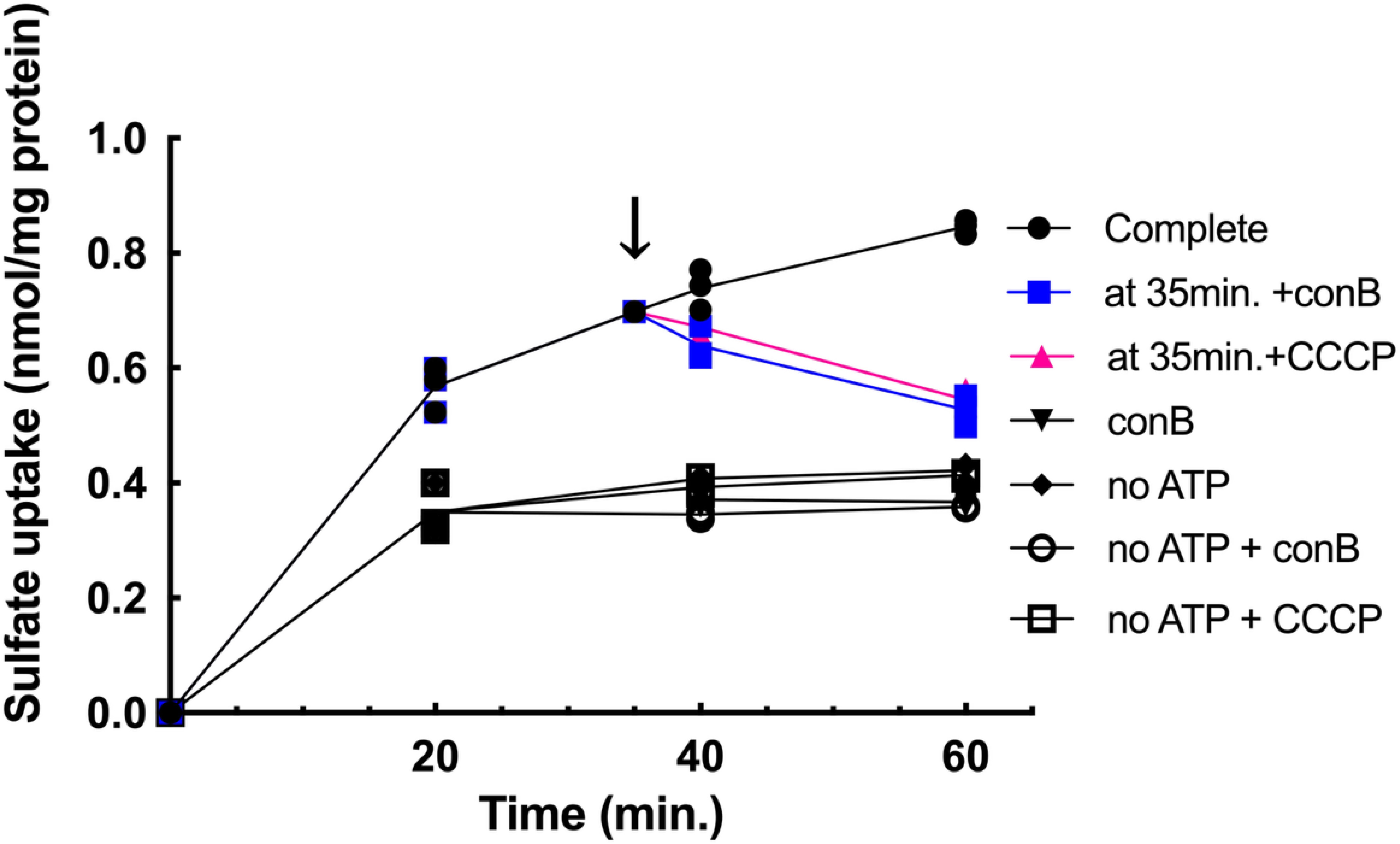

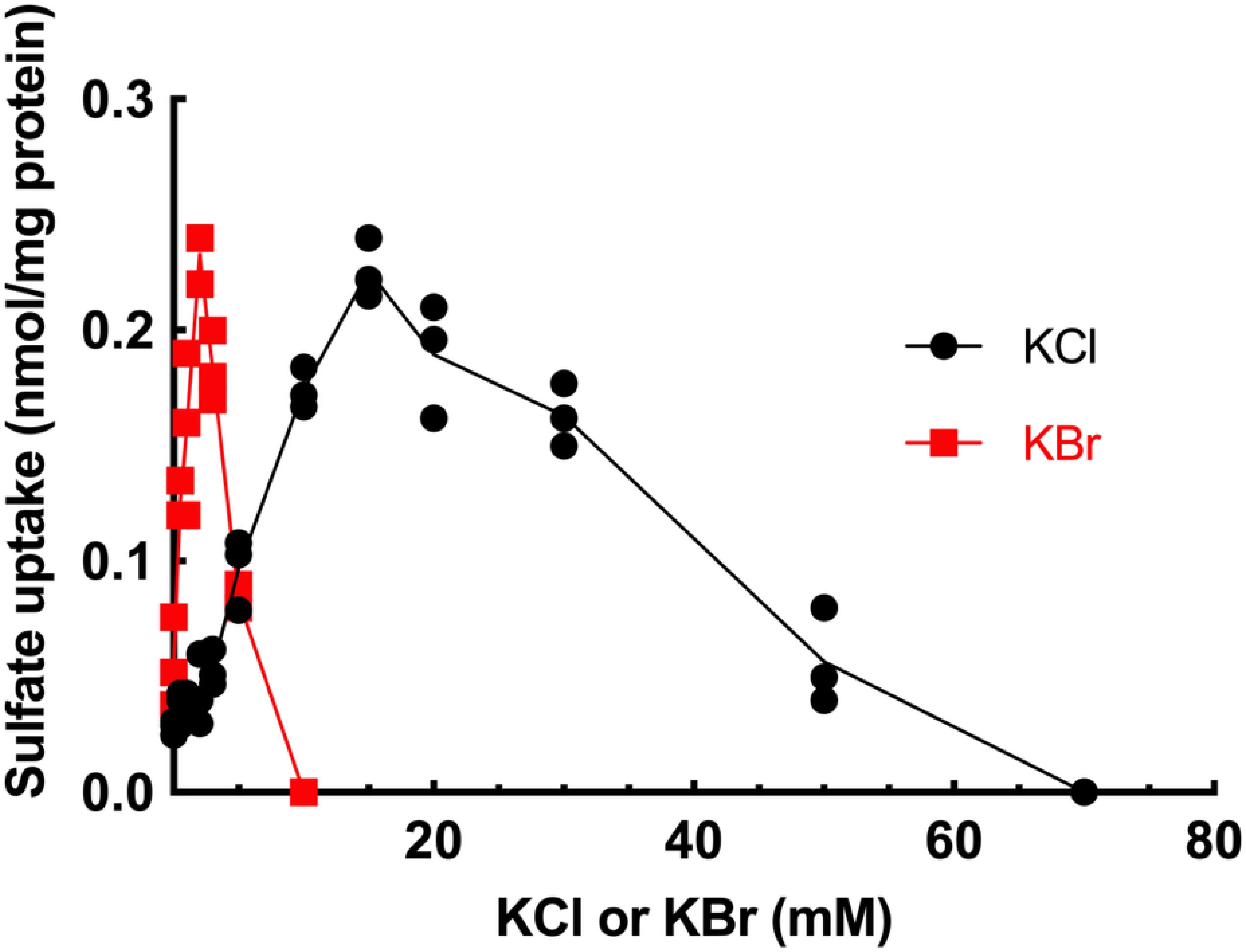


**Fig. S15 and S16. All points in Fig. 6A and 6D were plotted.**

**Fig.S15**

**Fig.S16**

16


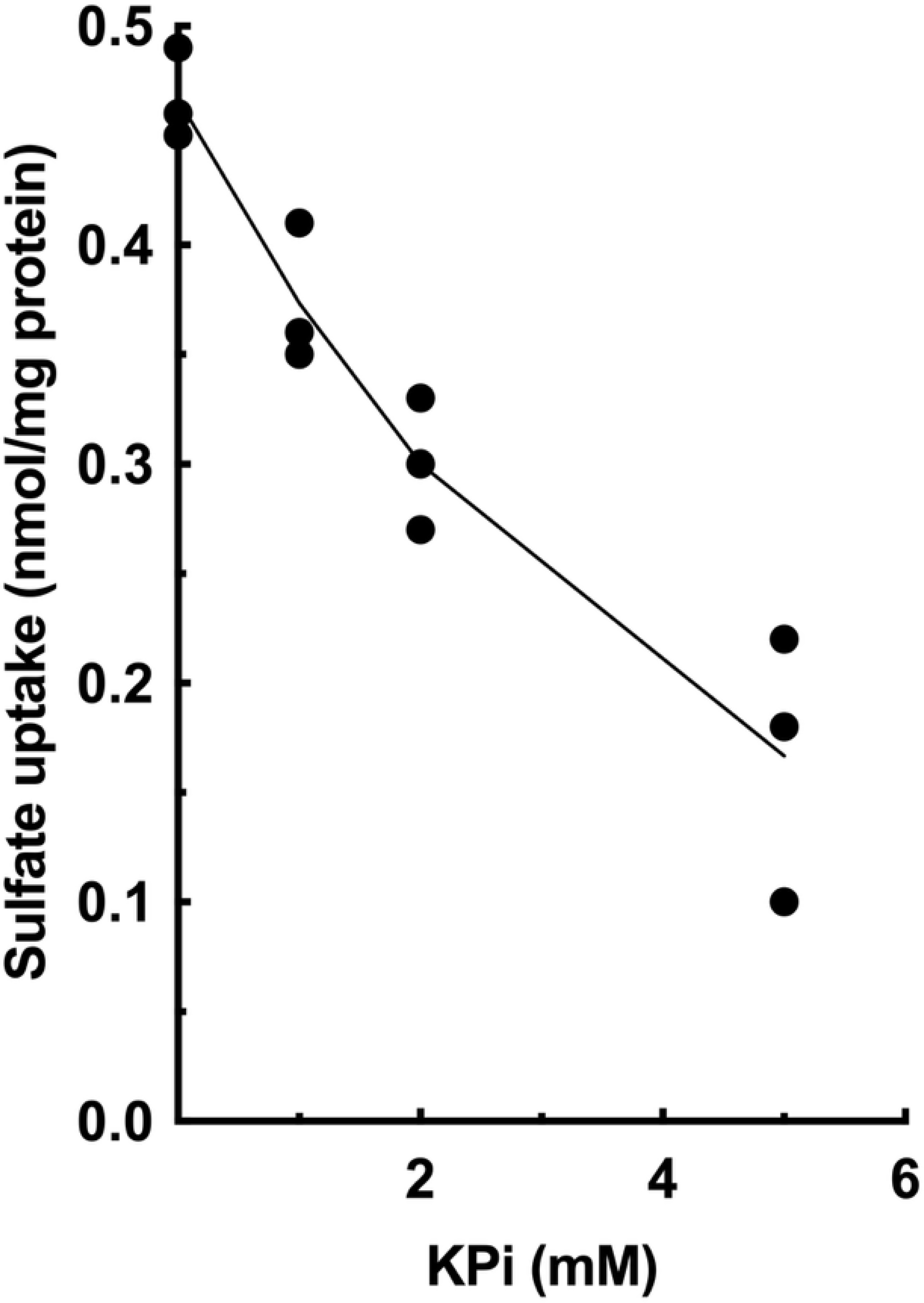

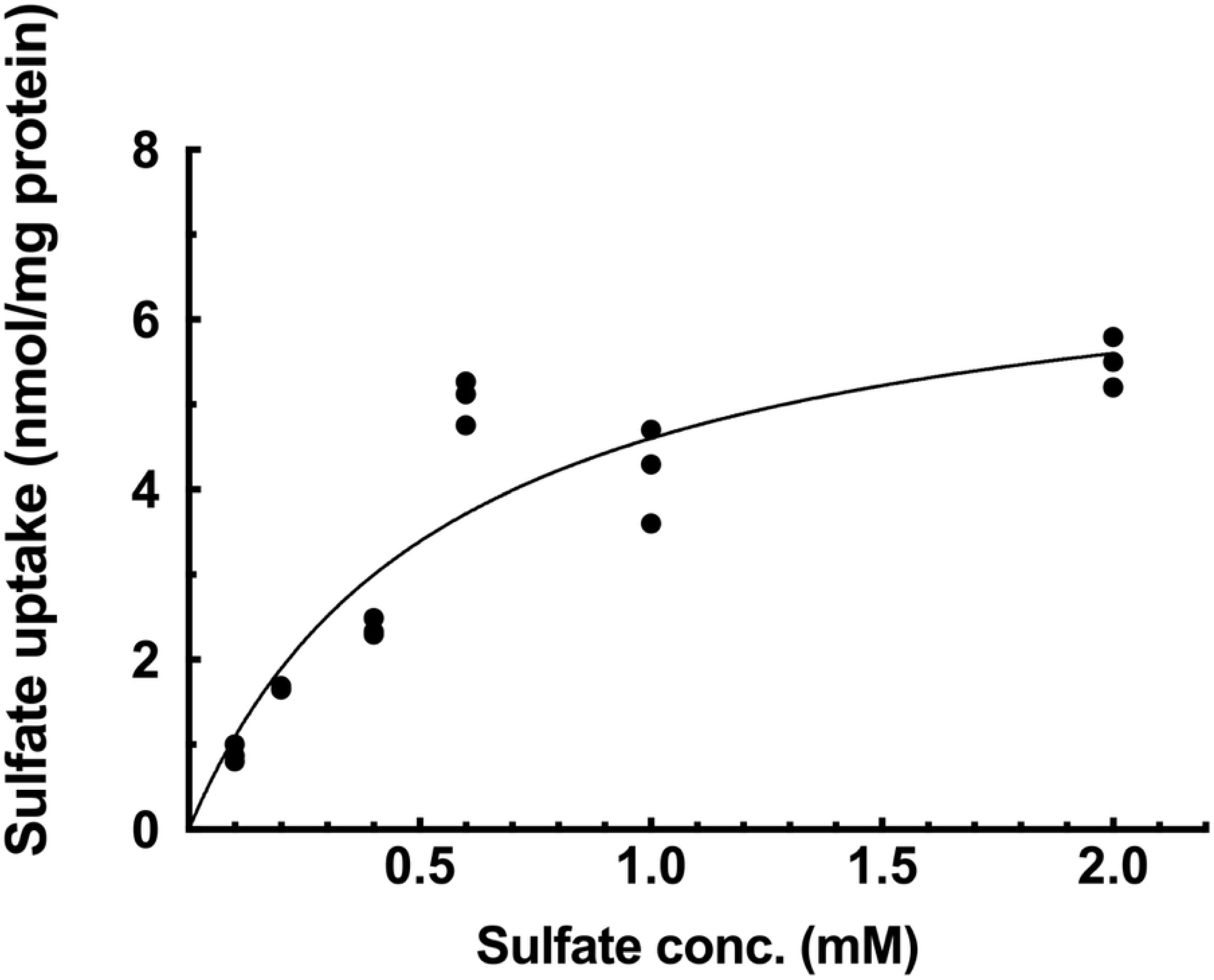


**Fig. S17 and Fig. S18. All points in Fig. 6E and Fig. 6F were plotted.**

**Fig.S17**

**Fig.S18**

17


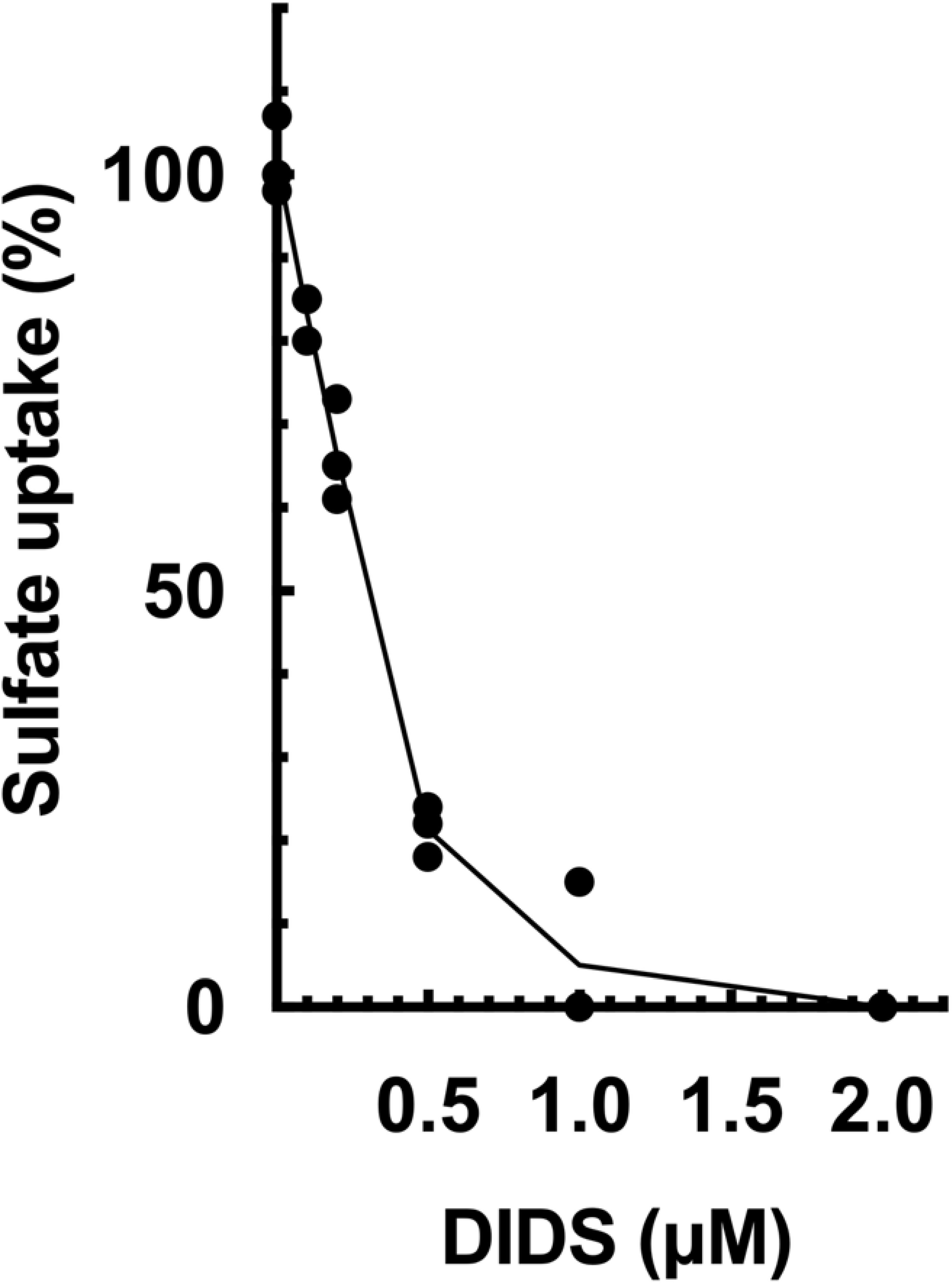

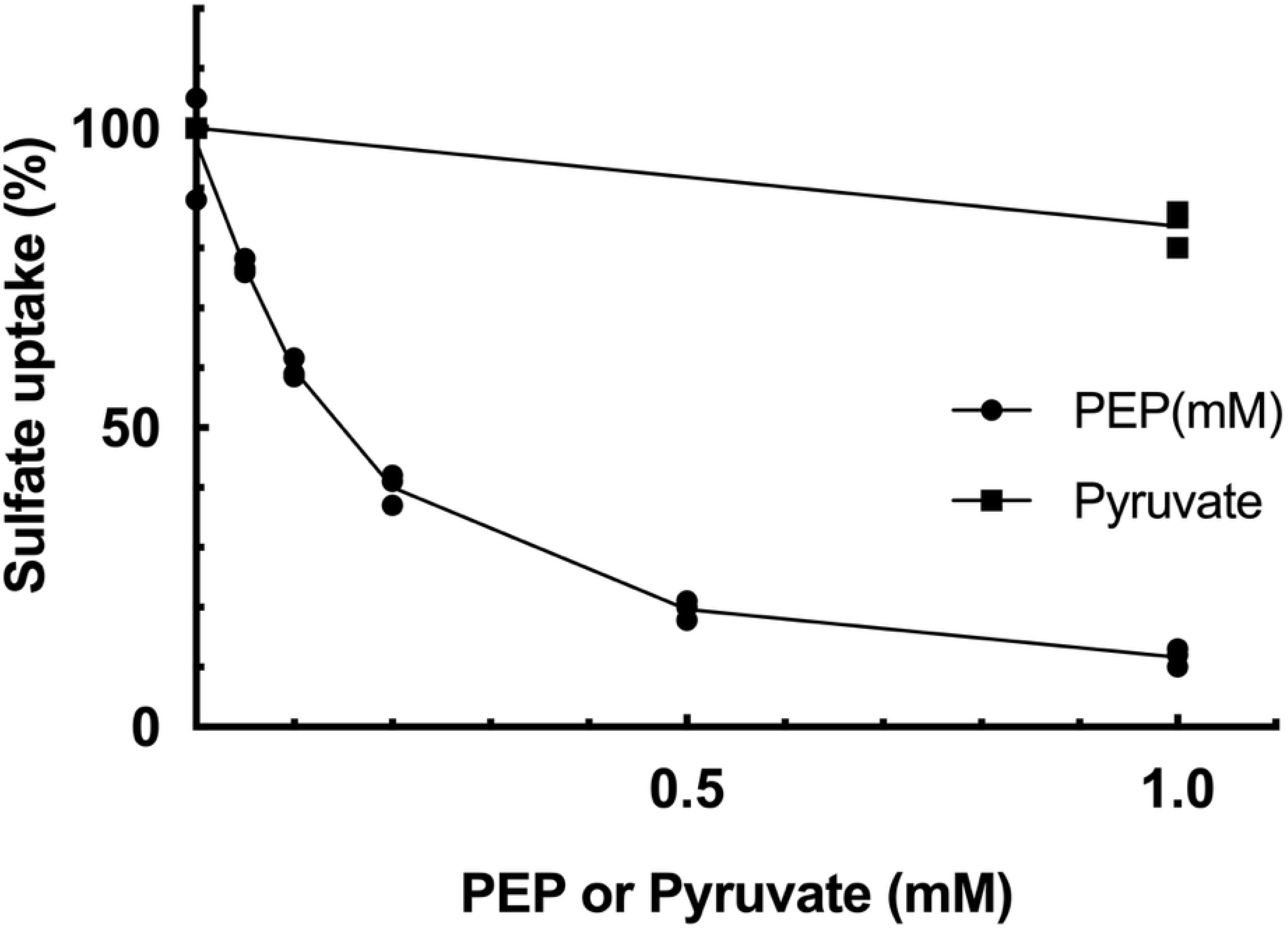


**Figs. S19 and S20. All points in Fig. 6H and 6I were plotted.**

**Fig.S19**

**Fig.S20**

18


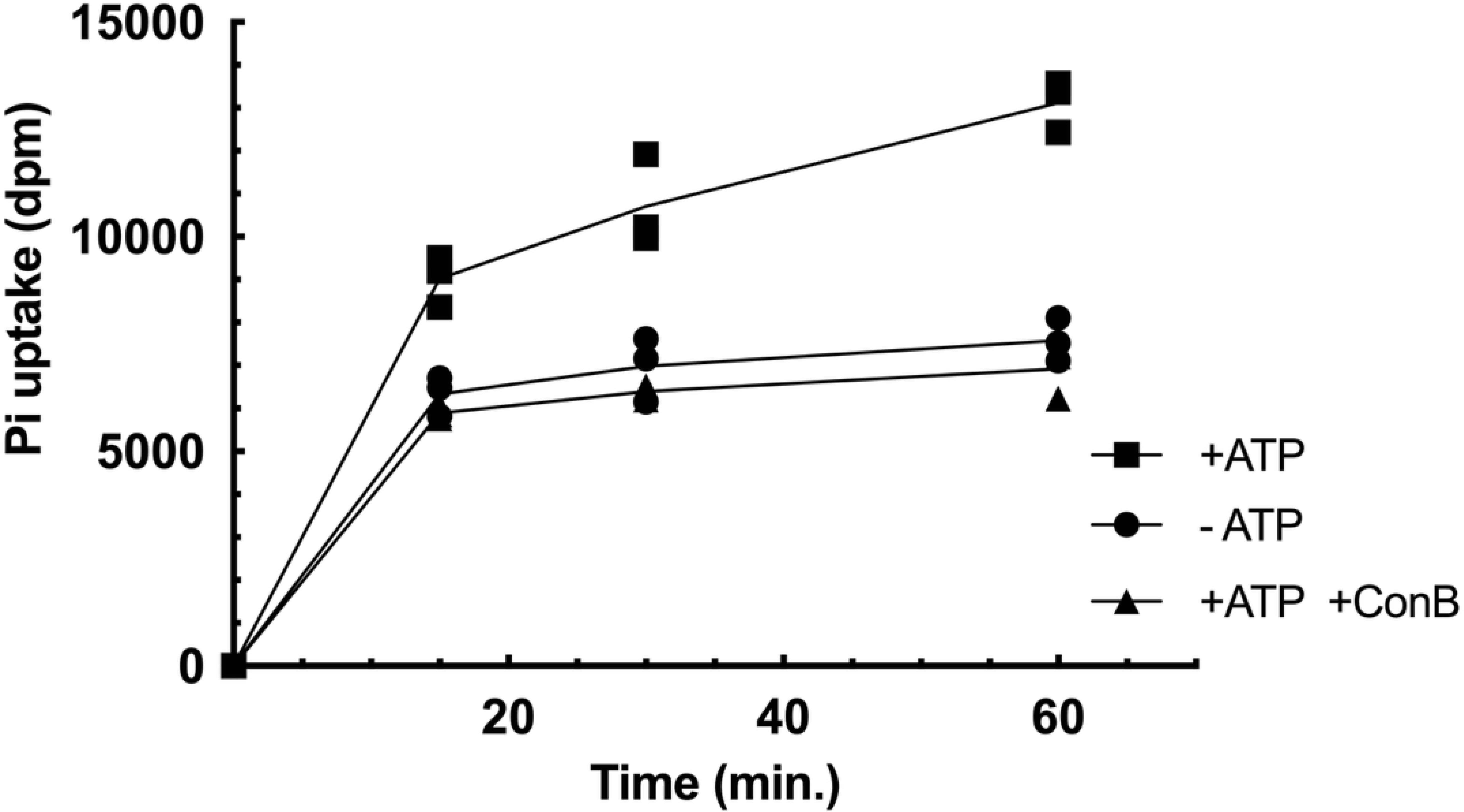


**Fig. S21. All points in Fig. 7A were plotted.**

19


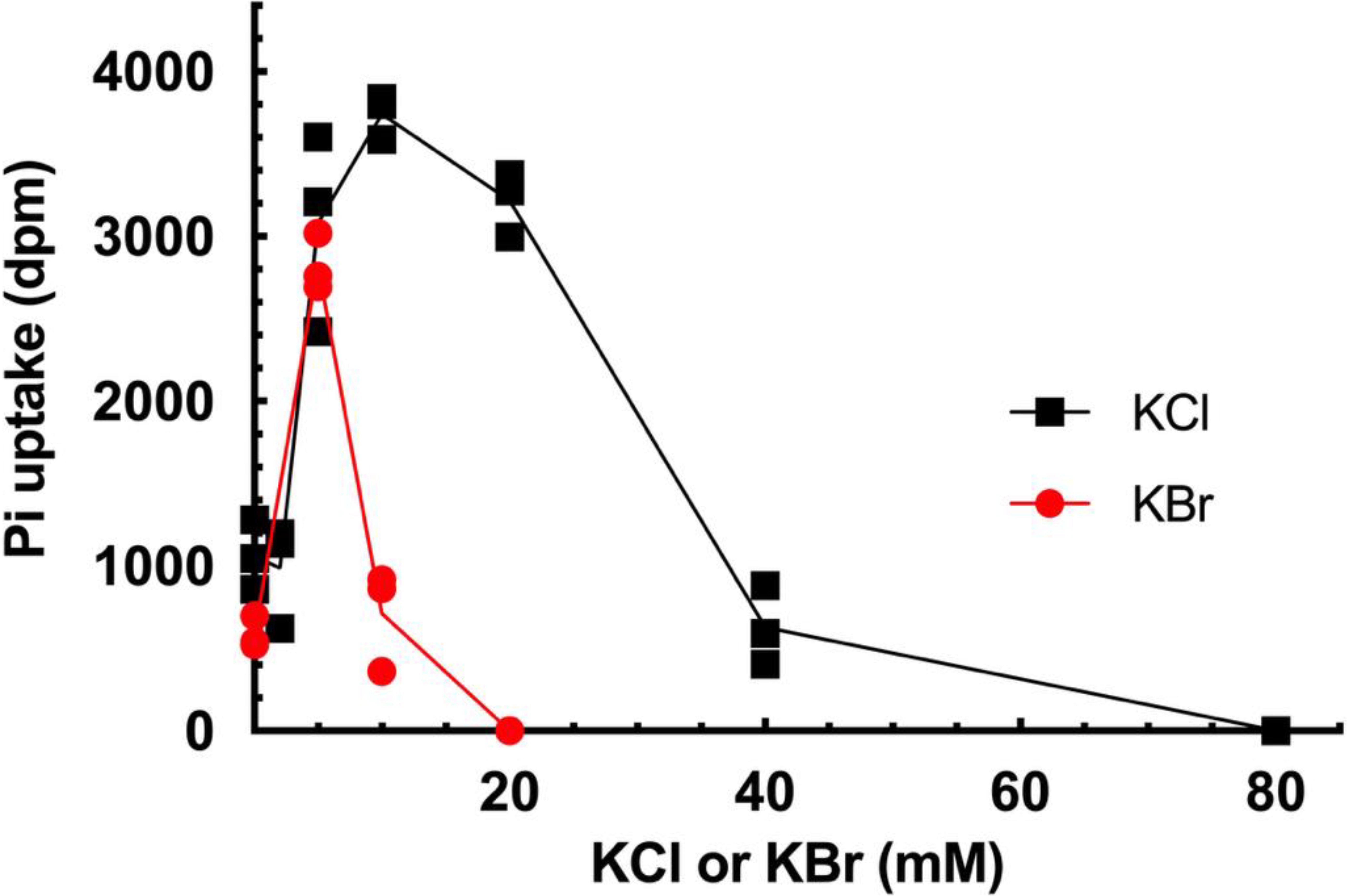

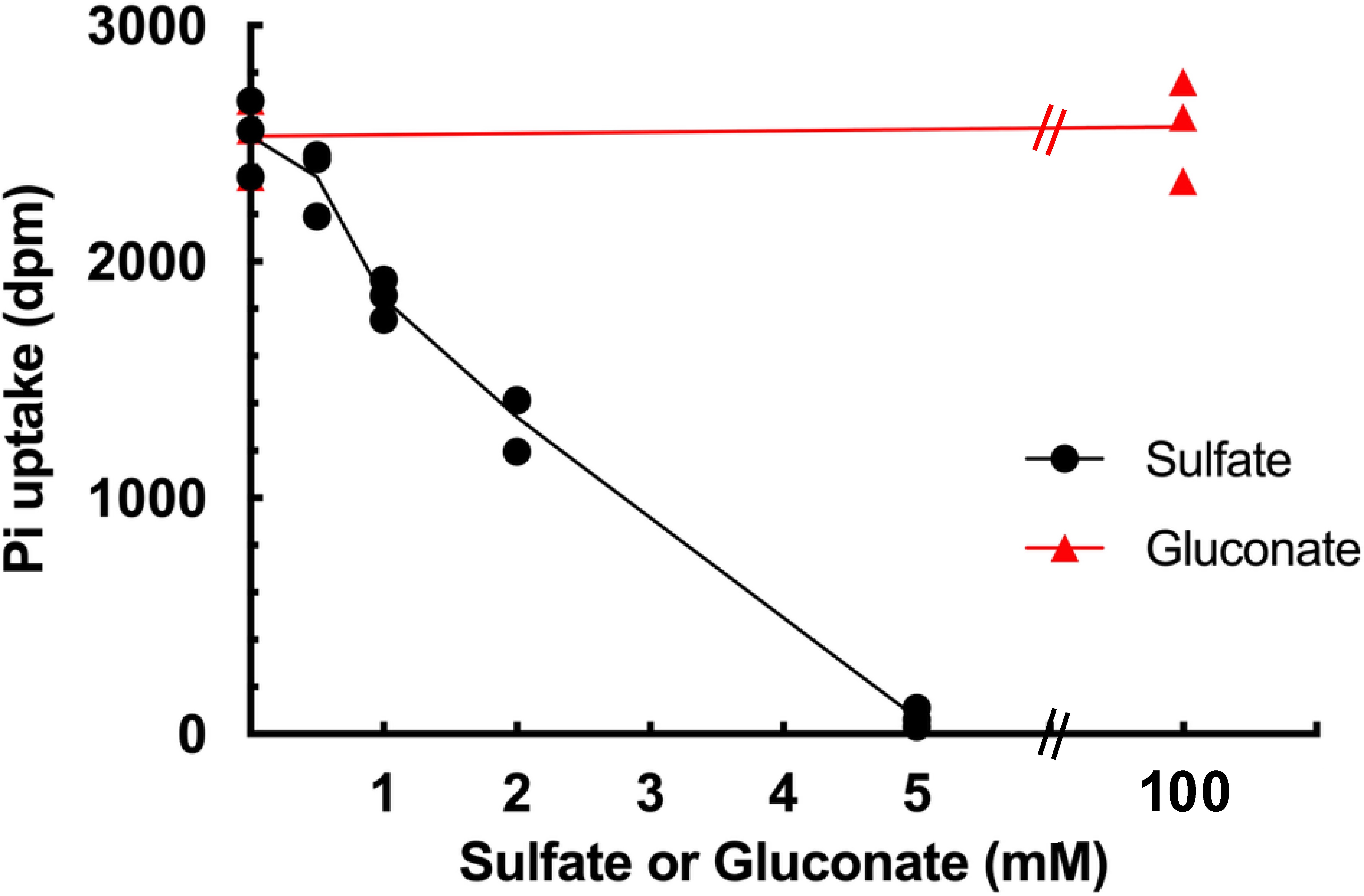


**Figs. S22 and S23. All points in Figs. 7D and 7E were plotted.**

**Fig.S22**

**Fig.S23**

20

**SUPPLEMENTAL REFERENCES**

1.

Leviatan, S., Sawada, K., Moriyama, Y**.**, Nelson, N. (2010) A combinatorial method for

overexpression of membrane proteins in E. coli. *J Biol Chem*. **285**:23548-23556.

2.

Moriyama, Y., Iwamoto, A., Hanada, H., Maeda, M., and Futai, M. (1991) One-step

purification of *Escherichia coli* H^+^-ATPase (F0F1) and its reconstitution into liposomes with neurotransmitter transporters. *J. Biol. Chem.* **266**, 22141-22146.

3.

Schaffner W and Weismann C. (1973) A rapid, sensitive, and specific method for the

determination of protein in dilute solution. *Anal Biochem* 56, 502-514.

4.

Iwai Y, Kamatani S, Moriyama S, Omote H. (2019) Function of essential chloride and arginine

residue in nucleotide binding to vesicular nucleotide transporter. *J Biochem* 165, 479-486.

21
